# Strategic utilization of chicken bones immobilized by *Aspergillus terreus* for sustainable bioremediation of cobalt: statistical modeling, kinetics and thermodynamic studies

**DOI:** 10.1186/s13036-025-00599-5

**Published:** 2026-03-11

**Authors:** Marwa Eltarahony, Eman H. El‑Gamal, Moustafa M. Salama, Amany Ibrahim

**Affiliations:** 1 Environmental Biotechnology Department, Genetic Engineering and Biotechnology Research Institute (GEBRI), City of Scientific Research and Technological Applications (SRTA-City), New Borg El‑Arab, Alexandria, 21934 Egypt; 2 Land and Water Technologies Department, Arid Lands Cultivation Research institute (ALCRI), City of Scientific Research and Technological Applications (SRTA-City), New Borg El‑Arab, Alexandria, 21934 Egypt; 3 Mathematics and Statistics Department, College of Science, Taif University, P.O. Box 11099, Taif, 21944 Saudi Arabia; 4 Department of Computer-Based Engineering Applications, Informatics Research Institute, City of Scientific Research and Technological Applications (SRTA-City), New Borg El‑Arab, Alexandria, 21934 Egypt; 5 Botany Department, Faculty of Women for Arts, Science and Education, Ain Shams University, Cairo, Egypt; 6 Department of Biology, College of Science, Taif University, P.O. Box 11099, Taif, 21944 Saudi Arabia; 7 High Altitude Research Center, Taif University, P.O. Box 11099, Taif, 21944 Saudi Arabia

**Keywords:** Biosorption, Wastewater treatment, Agricultural residues, Heavy metals, RSM, Immobilization, Hydroxyapatite, Filamentous fungi

## Abstract

To address the environmental and health risks of cobalt, agricultural byproducts (peanut shells and luffa sponge), spent chicken bones, and sea sand were recruited as immobilization matrices for *Aspergillus terreus*. Their adsorptive performance toward cobalt (250 mg L^−1^ at pH 7 and 30 °C for 1 h) was evaluated. The fungi-immobilized chicken bones (*At*-I-CB) showcased superior adsorptive performance (45.42±0.66%) among other examined bio-carriers. To maximize biosorption efficacy, Box–Behnken design inferred that 8.7 g L^−1^ of *At*-I-CB could adsorb 103.04 mg L^−1^ of cobalt by 96.8% at pH 6.76 within 101.4 min. Characterization via EDX, FTIR and SEM confirmed the presence of signals and functional groups of the hybrid constituents with higher distribution of cobalt particles on the surface of fungal conidia/hyphae and hydroxyapatite, which implied the structural heterogeneity/porosity of bio-carrier. Such design furnished a potent stabilization and enhanced the adsorption performance. Meanwhile, kinetic analysis declared that cobalt biosorption onto *At*-I-CB followed chemisorption mechanisms via electrostatic interactions, ion exchange, and complexation. The most fitting model was Intra-Particle Diffusion (R^2^ = 0.99), followed by Fractional Power and Elovich (R^2^ = 0.96), reflecting multi-stage dynamics of adsorption including external surface interaction, internal pore diffusion, and boundary layer diffusion. Thermodynamic studies revealed maximum adsorption capacity (84.60%) at 75 °C, with positive ΔG° values unveiling a non-spontaneous process with favorability at higher temperatures. Further, enthalpy change (∆H°; 49.54 kJ/mol) emphasized the endothermic chemisorption. Remarkably, no study reported such a deeply detailed bio-adsorption capacity of novel *At*-I-CB. Finally, this study demonstrates the feasibility of biowastes recyclability and safe utilization as natural, sustainable and eco-benevolent fungal carriers for implementing sustainability goals and circular bioeconomy.

**Supplementary Information:**

The online version contains supplementary material available at 10.1186/s13036-025-00599-5.

## Introduction

Global freshwater scarcity, aggravated by uncontrolled pollutants discharge into water bodies, has boosted research wheel into sustainable remediation techniques. Although chemical adsorbents are commonly used for heavy metals removal, bio-based materials are considered as a greener, cost-effective, and eco-friendly biosorbents [1–7]. Hereupon, advances in green technology are developing several environmentally acceptable surrogates for water purification [8]. Among them, filamentous fungi that proved their effectiveness in bioremediating various pollutants in wastewater, such as hydrocarbons [9], synthetic dyes [10], pharmaceutical byproducts [11], and heavy metals [12]. Filamentous fungi are characterized by unique compositions of their cell walls, which contain proteins (~60%), a lipid double layer rich in phospholipids and sterols (~40%), and various functional groups [13, 14]. This structure not only facilitates strong biosorption of metal ions but also enhances fungal tolerance against considerable metal concentrations under different environmental stresses [13, 15]. Additionally, the interconnected hyphal network that ranges from millimeters to centimeters enables fungi to be produced on a massive scale using inexpensive economic growth media and eco-friendly biosorbents, compared to other microorganisms [16]. Importantly, they can be utilized in both live and dead forms; the live cells facilitate active sorption (intracellular accumulation), while inactivated (dead) biomass enables passive adsorption via surface binding [17]. Remarkably, dead fungal biomass often outperforms live cells due to its superior desorption efficiency, reusability, reproducibility, simplified modeling and analysis, and lack of nutrient or growth media requirements. This approach reduces energy demand, causes no cell disruption, and minimizes the risk of mycotoxin dissipation; hence, it is a more economical and safer option for applications [18, 19].

Nevertheless, the utilization of free fungal biomass in bioremediation faces limitations such as low mechanical strength, biomass dilution, and washout risks [20]. To overcome these challenges, carrier-immobilized-fungal systems have gained momentum for pollution bioremediation, by enhancing cell resilience to environmental stressors, while maintaining metabolic activity/survivability, facilitating microbial separation, reusability, and increasing pollutant elimination efficiency [18]. Interestingly, immobilization techniques fall into two categories, namely, chemical (i.e., cross-linking) and physical (i.e., entrapment and surface adsorption), with latter being more common. Carriers include natural polymers such as Κ-carrageenan, diatomite alginate, and chitosan, while synthetic ones such as polysulfone, polyvinyl alcohol, and polyacrylamide that entrap and stabilize the microbial cells with many functional groups [21–23]. However, synthetic carriers often suffer from restricted sorbate diffusion rates, limited biomass-sorbent contact, and instability at low pH, as well as insufficient spaces and inappropriate mechanical strength to house active cell growth [18, 19]. In addition, biocompatibility, toxicity, non-biodegradability, market availability, and cost-effectiveness are also taken into consideration [23]. These difficulties could be surmounted by carriers’ surface immobilization of the active cells through physical adsorption process, which could be implemented by adhesive forces such as ionic interactions, Van der Waals forces, and hydrogen bonding. Notably, biomass functional groups and porous or fibrous immobilization matrices, accelerate the removal rate of pollutants through facilitating free access of the pollutant to sorption sites. Broadly, the physical adsorption immobilization technique is characterized by simplicity, efficiency, eco-friendliness, safety, and cost-effectiveness without the need for chemical additives as in the cross-linking method, which may cause toxicity for the immobilized active cells and the environment [20]. However, the support materials’ properties and their affinity are the decisive parameters that manage the success of the immobilization process. While the chemical, mechanical and thermal stability of the support materials, in addition to the environmental variations, are also vital [21]. Besides, the hydrophilic/hydrophobic nature of the microbial cell surface also participates in guaranteeing the strong binding of the cells on the substrates. Actually, the weak attachment forces involved in microbe-support material interaction may lead to elevate the leaching rate of the adsorbed cells away from the supporting matrix, eventually reducing the bioremediation rate.

Hence, the better selection of a convenient supporting matrix with potent adsorptive affinity while ensuring microbial functionality at a reasonable price is deemed as a logical step. In this regard, the investment of biological waste (e.g., agricultural byproducts and animal-derived biowastes) and their conversion to high-value-added materials, namely, sustainable immobilization supporting material, is deemed as alternative effective solution. Lignocellulosic agricultural residues and spent animal bones wastes from poultry industry have been widely recognized as inexpensive, abundant, and easily available porous nature bio-matrices suitable for use as immobilizing carriers [24]. Intriguingly, their fibrous network structure and natural functionality make them ideal candidates for sustainable applications. Notably, recyclability and safe utilization of such biowastes with fibrous network structure, as natural, could possibly contribute to a sustainable circular bioeconomy through waste management and energy conservation without the emission of incineration byproducts that adversely influence the environment [18, 24, 25]. Meanwhile, the adsorptive traits of these agro-industrial wastes were tremendously recruited for remediating heavy metals, dyes, antibiotics, and other organic contaminants by the dint of their functional characteristics and high surface area [26, 27].

Cobalt (Co^2+^) as a naturally occurring heavy metal (atomic number 27, density of 8.9 g cm^−3^), is essential in trace amounts as a component of vitamin B12 [28], although it exhibits toxicity even at low dosages [29]. Remarkably, its physicochemical traits enabled its anthropogenic usages in the manufacturing of hard metals, including steel production, and catalyst in the chemical and petroleum industries [30, 31], pigment in the paints and ceramics industries [32], as well as medical applications for treating anemia and cyanide poisoning. However, industrial discharges introduce Co^2+^ into water bodies at levels ranging from 60 to 6000 mg L^−1^ [33], exceeding natural background concentration in the surface and ground water (0.8 µg L^−1^) [34]. Contamination from mine runoff (0.9 mg L^−1^) [35] as well as household products contributing 1.36 μg L^−1^ and 0.86 μg L^−1^ in graywater and blackwater in Swedish homes, respectively [36]. High Co^2+^ levels (≥5 mg L^−1^) can cause various health symptoms such as asthma, heart failure, and thyroid and liver damage [37, 38]. Furthermore, cobalt may be carcinogenic [34, 39] and cause genetic abnormalities in living creatures [34, 40, 41]. While in agriculture, higher doses can reduce seedling growth, cause chlorosis, and decrease crop yields [29]. Although the World Health Organization (WHO) has not set a drinking water standard, it enforces a 0.05 mg L^−1^ limit for irrigation water [42, 43].

Accordingly, this study aimed to examine and compare the adsorptive capacity of dead *Aspergillus terreus* biomass, either free or immobilized on different matrices derived from agro-residues and spent chicken bones. Thereafter, statistical modeling (i.e., Box-Behnken design (BBD)) was employed to maximize the adsorption performance of the most efficient fungal-immobilized matrix. In addition, kinetics, and thermodynamic studies were also evaluated to ascertain a better and more detailed understanding of the adsorption mechanism. Such comprehensive details of novel hybrid system of *A. terreus* -immobilized biowaste were not studied before, till our knowledge.

## Materials and methods

### Fungal biosorbent, culture conditions and biomass preparation

*A. terreus* was screened and isolated from soil samples in an industrial region located in Saudi Arabia. The isolated fungus was inoculated on potato dextrose agar (PDA) with the following composition: 20 g Dextrose, 4 g Potato extract, and 15 g Agar in 1000 mL of distilled water. The plates were incubated for 7 days at 28 °C. The isolate was selected based on its highest biosorption capacity, morphological characterization, and molecular identification based on 28S-rRNA sequencing. The nucleotide sequence of the 28S-rRNA amplicon was submitted to GenBank to emphasize its identity and similarity with closely related species under the accession number of OQ275244. For preparing fungal biomass, 10 mL of spores’ suspension was inoculated in a 500 mL Erlenmeyer flask containing 150 mL of PD broth medium; this mixture was incubated at 28 °C for 72 h with vigorous stirring at a speed of 150 rpm. Thereafter, the culture pellets were filtered through a Whatman No. 4 filter paper to collect the fungal beads. The biomass was thoroughly washed with deionized water several times before weighing to eliminate any media residues. Finally, the fungal biomass was oven-dried at 90 °C for 48 h to obtain dead biomass, followed by milling and maintaining in an amber dry bottle at room temperature until use [44].

### Carrier’s matrices preparation and fungal immobilization

Herein, the peanut shells (PS) (*Arachis hypogaea*) and luffa sponge (LS) (*Luffa aegyptiaca*) were used as paradigms of lignocellulosic agro-wastes, while chicken bones (CB) and sea sand (SS) were used as hydroxyapatite- and siliceous-based immobilization matrices, respectively. All examined biowastes obtained from the local market were rinsed several times thoroughly with distilled water to remove any impurities or organic debris. Followed by oven drying at 60 °C for 10 h. Then, all examined materials were crushed, mechanically ground, and sieved for obtaining particles with approximate sizes between 6 and 10 mm [45]. For preparing the fungal-immobilized biowastes, about 0.5 g of each sterile examined matrix was added to 75 mL of sterile PD broth in 250 mL flasks that were previously inoculated with 5 mL of fungal spore suspension. The inoculated flasks were incubated in an orbital shaker incubator at 28 °C for 72 h. After incubation, the fungal-immobilized matrices were subjected to washing, then autoclaving at 121 °C for 20 minutes to ensure complete fungal inactivation. Thereafter, the sterilized carriers were oven-dried at 60 °C for 24 h.

### Characterization of fungal-immobilized matrices by scanning electron microscopy (SEM)

To visualize the overall morphology and texture differences of the examined matrices before fungal immobilization, as well as the uniform distribution of fungal hyphae on these matrices after immobilization, SEM (JSM 6360LA, JEOL, Akishima-shi, Japan) was employed. In addition, the cobalt-remediated samples were also visualized. All the samples were fixed in 2.5% glutaraldehyde overnight at 4 °C, followed by dehydration step with a gradient ethyl alcohol series (20:100%) for 15 min. The dried samples were mounted on copper stubs and coated with gold using a Polaron SC7620 Sputter Coater [46].

### Cobalt biosorption process

The cobalt biosorption experiments were carried out by comparing the adsorptive performance of the free dead fungal biomass and all prepared carriers in both cases (i.e., before and after fungal immobilization). About 0.25 g of each examined sample was evenly mixed in 100 mL of Co(NO₃)₂.6 H₂O solution (250 mg L^−1^) at neutral pH. All trials were conducted in triplicate and incubated at 30 °C for 1 h. At the end of the biosorption experiment, the concentrations of residual uncoupled Co^2+^ ions were detected quantitatively in the filtered solution by the inductively coupled plasma mass spectrometry (ICP-OES) Spectrometer (model Perkin Elmer Optima 2000DV) [47]. For calculating the adsorption efficiency, the following equation (Eq. 1) was applied. All required statistical analyses were performed using GraphPad Instat software (GraphPad Software, Version 6.0, Inc., La Jolla, CA, USA) based on the one-way ANOVA test, with the significance level of *p* < 0.05.

1 $$R = \left( {{{{C_0} - {C_e}} \over {{C_0}}}} \right)*100$$

Where: R (%) is the proportion of metal removed by the fungi, C₀ is the starting cobalt concentration (mg L^−1^), and C_e_ is the equilibrium final cobalt concentration (mg L^−1^).

On the other hand, the biosorption capacity that was used to calculate the amount of adsorbed Co^2+^ per mass of adsorbent (q) was denoted by Eq. (2).

2 $${q_e} = \left( {{{{C_0} - {C_e}} \over M}} \right)*V$$

q_e_ (mg g^−1^) is the biosorption capacity at equilibrium, V is the working volume for each batch test (L), and M is the mass of the adsorbent (g).

### Maximizing Co^2+^ biosorption of fungal-immobilized chicken bones via Box-Behnken design

As deduced from the previous step, fungal immobilized-chicken bones (*At-*I-CB) showed superior Co^2+^ adsorption performance. Subsequently, all further stages of the study would be executed by it. Remarkably, each 1 gm of *At-*I-CB contained 0.5 ± 0.07 gm of chicken bones and 0.43 ± 0.075 gm of fungal biomass. Initially, to maximize the adsorptive performance of this hybrid, BBD was applied. The experimental design includes 29 runs, were executed in 100 mL volume in triplicate for each run, at three coded levels: low (−1), medium (0), and high (+1), with 5 replicates at each level’s center coded values. Table 1 showed the examined factors aligned with their actual experimental values. The derived second-order polynomial equation is provided as follows (Eq. 3):

3 $$Y = {\beta _0} + \sum\limits_{i = 1}^4 {{\beta _i}{X_i}} + \sum\limits_{i = 1}^4 {{\beta _{ii}}X_i^2 + \sum {\sum\limits_{i < j = 2}^4 {{\beta _{ij}}{X_i}{X_j} + \varepsilon } } } $$

**Table 1:** The design of the 29 Box-Behnken runs of Co^2+^ ions removal by *At*-I-CB hybrid system at three coded levels of independent variables, their experimental values, experimental/predicted Co^2+^ biosorption percentage and studentized residuals

Run	*X*_*1*_	*X*_*2*_	*X*_*3*_	*X*_*4*_	Removal efficiency		
					Actual Co^2+^ biosorption	Predicted	Residual
	pH	*At*-I-CB weight	Contact Time	Co^2+^ Concentration			
		(g L^−1^)	(min)	(mg L^−1^)	(%)		
1	5.5	5.5	67.5	187.5	54.83	55.37	−0.5454
2	8	5.5	67.5	300	17.13	19.10	−1.98
3	5.5	5.5	120	300	45.65	45.12	0.5252
*4*	3	5.5	67.5	75	60.72	61.56	−0.8341
5	5.5	10	15	187.5	19.25	19.80	−0.5456
6	3	1	67.5	187.5	10.26	8.72	1.54
7	3	5.5	120	187.5	20.91	21.49	−0.5731
8	5.5	1	15	187.5	13.93	15.55	−1.62
9	5.5	1	67.5	300	21.95	20.75	1.21
10	8	10	67.5	187.5	42.51	41.72	0.7885
11	8	5.5	15	187.5	10.26	9.20	1.06
12	5.5	5.5	67.5	187.5	56.47	55.37	1.10
13	5.5	5.5	120	75	98.70	96.30	2.40
14	5.5	10	67.5	300	46.27	45.51	0.7546
15	3	10	67.5	187.5	28.04	27.12	0.9169
16	5.5	10	67.5	75	95.48	96.20	−0.7213
17	5.5	10	120	187.5	59.65	60.84	−1.19
18	8	5.5	67.5	75	95.85	98.23	−2.38
19	5.5	5.5	67.5	187.5	56.50	55.37	1.12
20	5.5	5.5	67.5	187.5	54.26	55.37	−1.11
21	5.5	5.5	67.5	187.5	54.80	55.37	−0.5672
*22*	8	5.5	120	187.5	48.16	47.06	1.10
23	5.5	5.5	15	300	15.37	15.45	−0.0766
24	8	1	67.5	187.5	21.65	20.23	1.41
25	3	5.5	15	187.5	8.04	8.66	−0.6190
26	5.5	1	120	187.5	22.93	25.20	−2.27
27	3	5.5	67.5	300	29.23	29.66	−0.4304
28	5.5	1	67.5	75	80.81	81.08	−0.2704
29	5.5	5.5	15	75	77.09	75.28	1.80

Where: ${\beta _0}$ is the intercept term (the model’s regression coefficient); ${X_i}$ and ${X_j}$are the independent variables; and $Y$is the expected response Co^2+^ biosorption %;${\beta _i}$, ${\beta _{ii}}$and ${\beta _{ij}}$are the linear, quadratic, and interaction effects, respectively; and $\varepsilon$is the experimental error.

### Statistical analysis and verification of the model

The statistical software (Design-Expert 13 Inc. software) was employed to construct experimental designs “matrices” and to perform further statistical analyses of BBD data (regression analysis and ANOVA). Moreover, the relationship between the biosorption percentage and the tested variables was depicted graphically by three-dimensional surface plots (3D) and two-dimensional contour plots (2D). In addition, the optimal levels of each examined variable were predicted to maximize Co^2+^ biosorption percentage. Under such predicted conditions, the model was verified by comparing the Co^2+^ biosorption percentage that predicted from BBD with the basal conditions.

### Confirming Co^2+^ biosorption on fungal-immobilized chicken bones under optimized conditions

The surface functional groups present in the *At-*I-CB, which substantially aid the Co^2+^ biosorption process, were identified via Fourier transform infrared spectroscopy (FTIR) analysis at a wavelength range from 3500 to 500 cm^−1^. Following Co^2+^ biosorption, the dehydrated *At-*I-CB matrix was pulverized into fine powder. The powdered sample was mixed with 1.0% KBr (w/w) and pressed into discs for FTIR analysis [47]. To further confirm the involvement of Co^2+^ within the *At-*I-CB matrix, elemental analysis was emphasized through an energy dispersive X-ray (EDX) analyzer coupled with scanning electron microscopy (SEM, JEOL JSM 6360LA, Japan). These microchemical analysis not only confirmed the presence of cobalt within the immobilized matrix but also allowed for the detection of other elements associated with *At-*I-CB matrix. More so, the morphological features were also captured via SEM, as mentioned previously [48].

### Kinetics and thermodynamic studies of Co^2+^ biosorption on fungal-immobilized chicken bones

Under the optimized conditions of each examined variable, a kinetic adsorption process was conducted to determine the maximum adsorption capacity and to investigate the adsorption mechanism of cobalt ions on the *At-*I-CB matrix. The kinetic experiment was carried out under optimized conditions that deduced from BBD, in flasks contained solution volume of 100 mL, at varying contact times ranging from 15 to 100 minutes, using a fixed initial concentration of 100 mg L^-1^, an adsorbent dosage of 8.7 g L^-1^, and a solution pH adjusted to 6.76. The mixture was shaken at 150 rpm. After each time, the remaining amount of Co^2+^ was determined as previously described. The amount of cobalt adsorbed at various contact times (qt) was calculated using the following eq. (4):

4 $${q_t} = \left( {{{{C_0} - {C_t}} \over M}} \right)*V$$

q_t_ (mg g^−1^) is the biosorption capacity at time T (min), and C_t_ is the Co^2+^ ions (mg L^−1^) amount at the same time, while Co, M, and V were mentioned previously in the equations (1 and 2). The kinetic reactions utilized numerous linear kinetic models as follows:

5 $$\mathrm{Fractional\;Power}\;{\bf{\it{ln }}}{{\bf{\it{q}}}_{\bf{\it{t}}}} = {\bf{\it{lna}}} + {\bf{\it{b lnT}}}$$

6 $$\mathrm{Elovich}\;{{\bf{\it{q}}}_{\bf{\it{t}}}} = {\bf{\it{\beta ln}}}\left( {{\bf{\it{\alpha \beta }}}} \right) + {\bf{\it{\beta lnT}}}$$

7 $$\mathrm{Pseudo-First\;Order}\;{\bf{\it{ln}}}\left( {{{\bf{\it{q}}}_{\bf{\it{e}}}} - {{\bf{\it{q}}}_{\bf{\it{t}}}}} \right) = {\bf{\it{ln}}}{{\bf{\it{q}}}_{\bf{\it{e}}}} - {{\bf{\it{k}}}_1}{\bf{\it{T }}}$$

8 $$\mathrm{Pseudo-Second\;Order}\;{\bf{\it{T}}}/{{\bf{\it{q}}}_{\bf{\it{t}}}}{\bf{\it{ }}} = \left( {1/{{\bf{\it{k}}}_2}{\bf{\it{q}}}_{\bf{\it{e}}}^2} \right) + \left( {1/{{\bf{\it{q}}}_{\bf{\it{e}}}}{\bf{\it{T}}}} \right)$$

9 $$\mathrm{Intraparticle\;Diffusion}\;{{\bf{\it{q}}}_{\bf{\it{t}}}} = {{\bf{\it{L}}}_{\bf{\it{s}}}} + {{\bf{\it{D}}}_{\bf{\it{s}}}}{\bf{\it{ }}}{{\bf{\it{T}}}^{0.5}}$$

Where qt and *q*_*e*_ are the adsorption capacity of Co^2+^ at time *T* (min) and the equilibrium (mg g^−1^), respectively. *a* is the Functional Power constant (mg g^−1^), and *b* is the adsorption rate constant (min^−1^); *α* and *β* are the initial sorption rate (mg g^−1^ min^−1^) and the adsorption constant (g mg^−1^) of the Elovich Model. The Pseudo-First-Order and Pseudo-Second-Order adsorption rate constants are *k*_*1*_ (min^−1^) and *k*_*2*_ (g mg^−1^ min^−1).^ *L*_*s*_ and *D*_*s*_ are the boundary layer thickness (mg g^−1^) and Intra-Particle Diffusion rate constant (mg min^−0.5^ g^−1)^ at the different adsorption stages, respectively.

To assess the thermodynamic parameters of Co^2+^ ions adsorption onto the *At-*I-CB matrix, the impact of temperature was investigated at seven temperatures between 15 °C and 75°C. The experiment was studied according to the optimized conditions. Thermodynamic parameters such as the enthalpy change (ΔH°), entropy change (ΔS°), and Gibbs free energy change (ΔG°) were analyzed to better understand the influence of temperature on the adsorption process. These parameters were determined using the Van’t Hoff graph by plotting ln *K*_*d*_ versus *1/T*, where the slope and the intercept provide ΔH° and ΔS°, respectively, using the thermodynamic equilibrium constant (*K*_*d*_, eq. 10) [49]. The following equations were employed to calculate the thermodynamic parameters related to Co^2+^ adsorption onto *At*-I-CB.

10 $${{\bf{\it{K}}}_{\bf{\it{d}}}} = {{{{\bf{\it{q}}}_{\bf{\it{e}}}}} \over {{{\bf{\it{C}}}_{\bf{\it{e}}}}}}$$

11 $${\bf{\it{\Delta G}}}^\circ = {\bf{\it{ \Delta H}}}^\circ - {\bf{\it{T\Delta S}}}^\circ {\bf{\it{ }}}$$

12 $${\bf{\it{\Delta G}}}^\circ = - {\bf{\it{RT}}}{\bf{ln}}{{\bf{\it{K}}}_{\bf{\it{d}}}}{\bf{\it{ }}}$$

13 $${\bf{\it{ln}}} {{\bf{\it{K}}}_{\bf{\it{c}}}} = {{{\bf{\it{\Delta S}}}^\circ } \over {{\bf{\it{RT}}}}} - {{{\bf{\it{\Delta H}}}^\circ } \over {{\bf{\it{RT}}}}} = {{{\bf{-\it{\Delta G}}}^\circ } \over {{\bf{\it{RT}}}}}$$

Where: *R* represents the universal gas constant (8.314 J/mol K), and *T* shows the absolute temperature.

## Results and discussion

### Characterization of fungal-immobilized matrices by scanning electron microscopy (SEM)

**Fig. 1:**
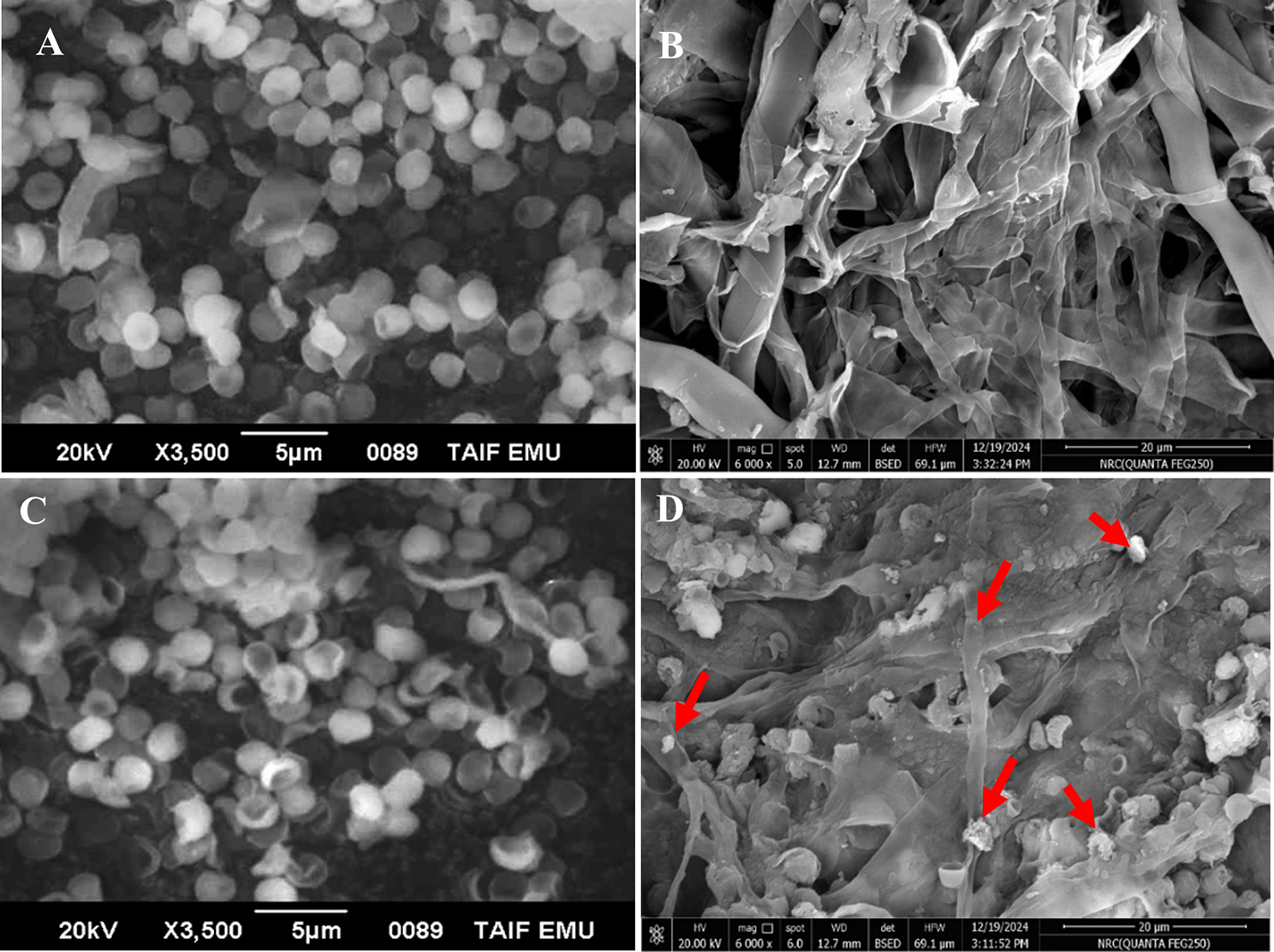
SEM micrographs representing *A. terreus* cells before and after Co^2+^ biosorption. **A**)- live conidia, **B**)-live hyphae, **C**)-dead conidia before Co^2+^ biosorption and **D**)- dead biomass after Co^2+^ biosorption. Red arrows symbolized Co^2+^ particles adsorbed on fungal surface

**Fig. 2:**
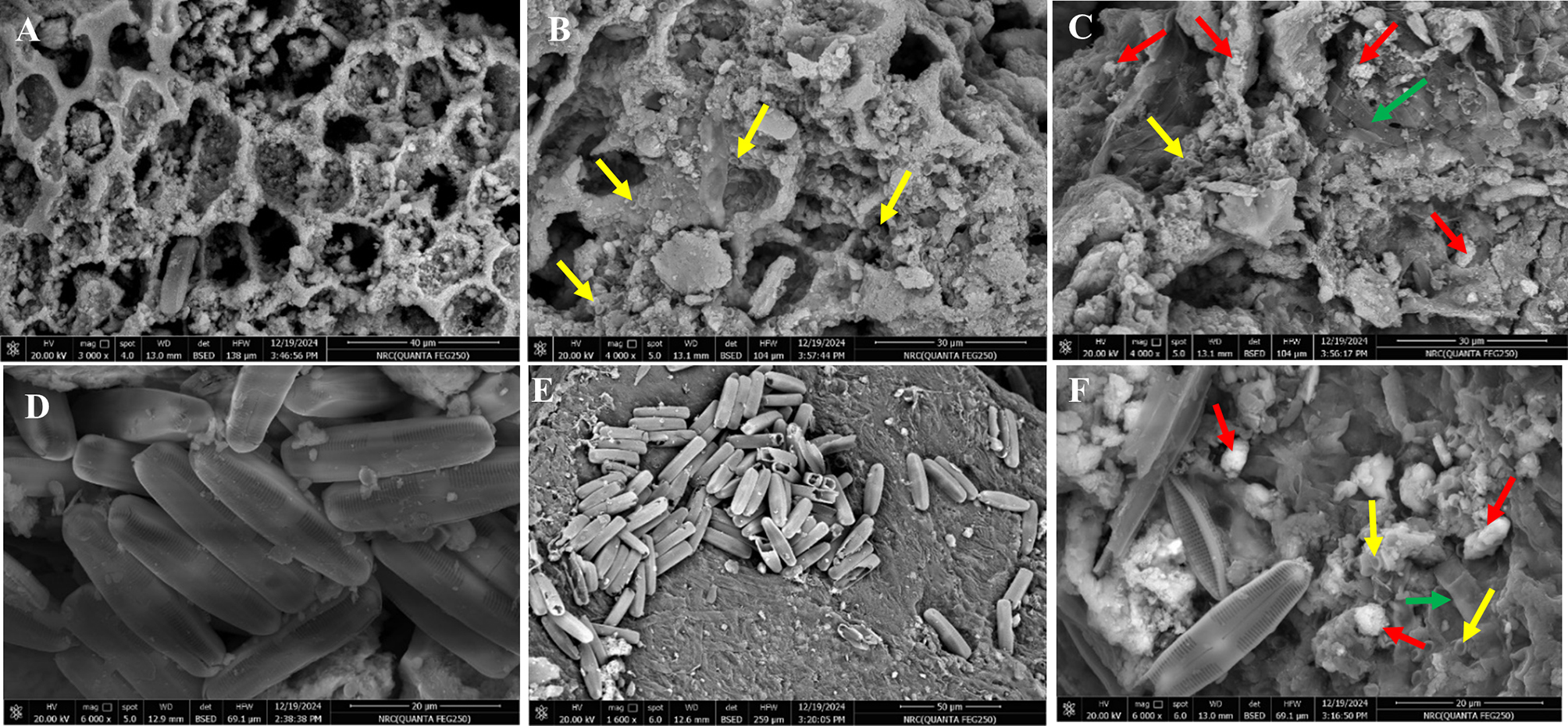
SEM micrographs of pristine chicken bones (CB) and sea sand (SS) before/after the immobilization with *A. terreus* cells and after Co^2+^ biosorption. **A**)- Pristine CB before immobilization, **B**)- Pristine CB after fungal immobilization, **C**)-*At*-I-CB after Co^2+^ biosorption **D**)- Pristine SS before immobilization, **E**)- Pristine SS after fungal immobilization and **F**)- *At*-I-SS after Co^2+^ biosorption. Red arrows symbolized Co^2+^ particles adsorbed on the surface, yellow arrows referred to fungal conidia and green arrows referred to fungal hyphae

**Fig. 3:**
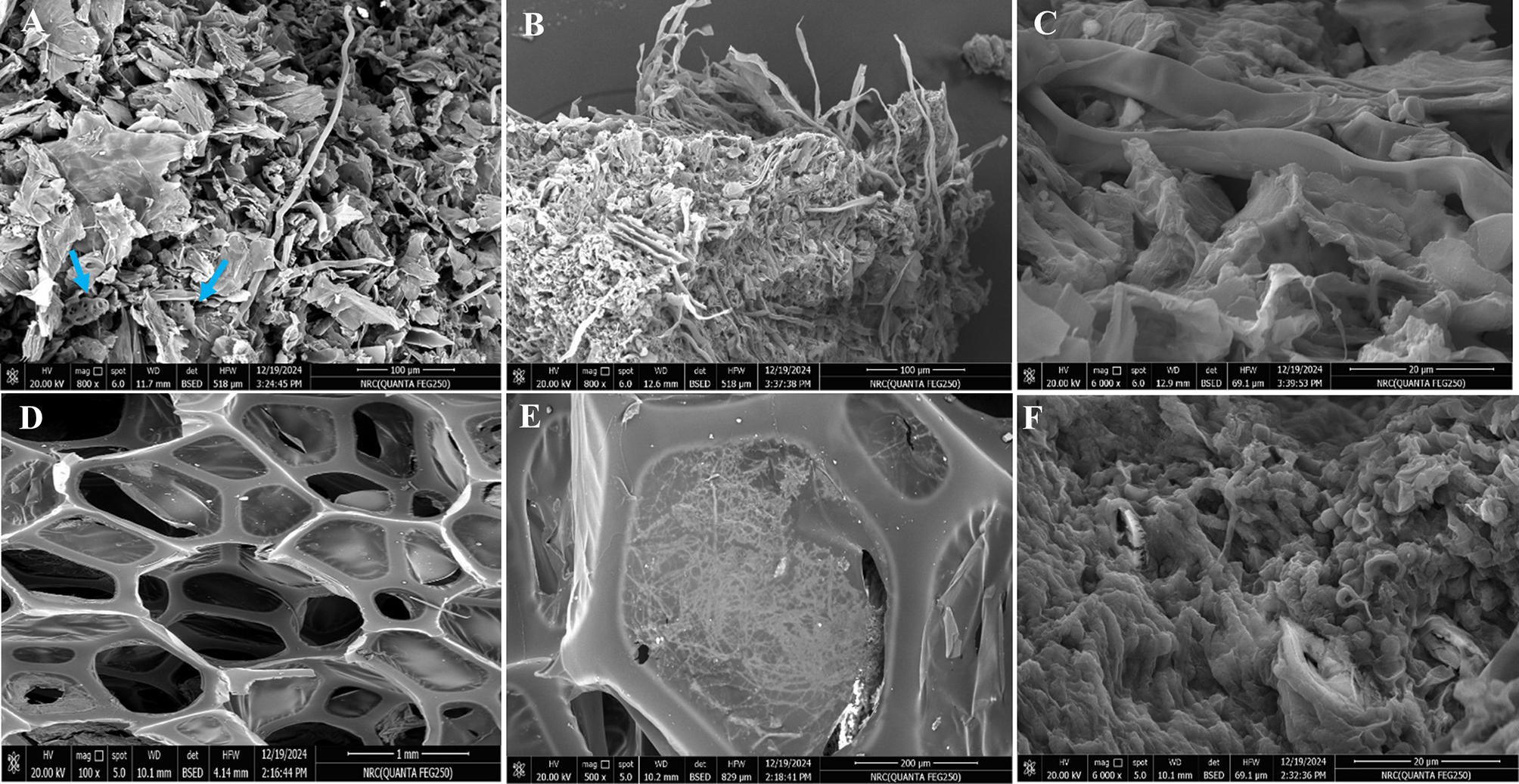
SEM micrographs of pristine peanut shell (PS) and luffa sponge (LS) before/after the immobilization by *A. terreus* cells and after Co^2+^ biosorption. **A**)- pristine PS before immobilization, **B**)- pristine PS after fungal immobilization, **C**)-*At*-I-PS after Co^2+^ biosorption **D**)- pristine LS before immobilization, **E**)- pristine LS after fungal immobilization and **F**)- *At*-I-LS after Co^2+^ biosorption. Blue arrows refer to particles containing fractures and holes

Essentially, to augment the surface area tailored for Co^2+^ biosorption, different biowaste matrices, which varied in their functional structures, were employed. As a representative of hydroxyapatite biowastes, chicken bones were used. While peanut shells and luffa sponge were utilized as carbonaceous matter derived from lignocellulosic agro-wastes. On the other hand, sea sand served as a siliceous-based carrier. It is worth mentioning that the reuse, recycling, or resource recovery of such solid biowastes as microbial carriers could be considered as a way for waste management. Wherein, the continuous production of agricultural crops and farm animals leads to the continuous release of both agro-wastes and spent animal bones in large volumes or quantities, which are non-biodegradable and occupy large spaces in the environment. Hereby, easily provide the provision of hosting surfaces for other pollutants, which are collectively symbolized as environmental burden [50–52]. However, by the dint of their nature, a proper management system could be manipulated easily. Interestingly, all examined biowastes in the current study were employed tremendously and successfully in remediating different types of contaminants in previous studies [27, 50, 53–55]. Wherein, the structural properties and chemical constituents of all examined materials made them highly attractive matrices for pollutants biosorption. Regarding lignocellulosic residues, they exhibit a three-dimensional fibrous network of cellulose, hemicellulose, and lignin, which are the main components with different percentages. Such hollow microfibers are porous and permit considerable adhesion between their surface and adsorbed material [26, 56]. Similarly, the spent animal bones waste derived from the poultry/animal industry show high surface area with multiple functional groups, which also foster the potent chelation of the pollutants on them [57]. While sea sand as a silica-based material possesses versatile surface chemistry and high mechanical strength with high porosity and wide pores enabling more contact with contaminants [58]. Additionally, the common features that characterize these biowastes are their ready availability, low capital cost, ecofriendly, biocompatibility, and renewability. Nonetheless, a plethora of studies reported the importance of surface treatment of such biowastes to increase their functionality and porosity, while enhancing their limited surface area. Chemical treatment with sodium hydroxide or organic acids is the widely applied method [26, 56], however, higher temperature describes thermal modification, and physical treatment could be exerted through ultrasonication and ultraviolet to generate active carbon or biochar forms [59–63]. Herein, all the utilized bio-carriers were investigated in their pristine state without any type of treatment (i.e., chemical, physical, or even thermal), which triggered the whole process economic and incontestable ecofriendly. Additionally, the current study addressed a dual task, which is heavy metal myco^-^remediation and simultaneously biowastes management. Both purposes meet unequivocally the sustainability goals and implement techno-economic productivity, which aligns with the top priorities of international environmental politics. Therefore, the success in immobilizing the fungal biomass on the examined matrices was confirmed firstly through SEM (Figs. 2 and 3). Initiating by the morphological features of the *A. terreus* cells, the conidia appeared healthy, globose to ellipsoidal with smooth-walled surfaces, ranging in size from 0.43 to 0.62 μm and clustered together while separated from their conidiophores (Fig. 1–A). Meanwhile, cylindrical-shaped mycelia with smooth surfaces appeared aggregated in a framework (Fig. 1–B). Such features are commonly observed in this strain as documented by [64, 65]. Upon drying to obtain the dead biomass, significant alteration in the surface morphology was vividly noticed (Fig. 1–C). However, Fig. 1–D depicted the dead biomass after the adsorption of Co^2+^. Wherein, Co^2+^ was adsorbed on the surface of conidia and hyphae, which became wrinkled in their surfaces. Notably, some bright spots of clumsy deposits were scattered randomly on the surface of the dead biomass (Fig. 1–D, red arrows), which could be assigned to the adsorbed Co^2+^. As reported by [66], the metal particles appeared brighter in the SEM micrographs owing to their higher atomic number relative to the biological material.

On the other hand, by combining the biowastes along with fungal growth medium, inoculation with spore suspension and incubation, a luxuriant fungal proliferation was observed on the entire surface of all examined biowastes, implying that all of them didn’t have any negative leverage on the fungal growth. Remarkably, the textural morphologies, surface roughness, and porosity of all examined biowastes were visualized prior and post to fungal immobilization and also after Co^2+^ adsorption via SEM (Figs. 2 and 3). Regarding CB, its surface demonstrated an observable distribution of the well-developed macroporous surface of a honeycomb-like assembly with a rough surface (Fig. 2–A). Such porosity and structural heterogeneity aid substantially in the adsorption performance and also the adsorption rate [67]. Similarly, the general morphology of the CB of the current study agreed with that found by [67], while resembling activated carbon-based CB obtained by [67, 68]. However, a considerable colonization of fungal cells was detected in the exposed areas and the inner surfaces of CB, reflecting the intrinsic role of rough and irregular surfaces with multiple cavities and edges in providing a large surface area for fungal adhesion (Fig. 2–B, yellow arrows). Concerning SS, its particles possessed an ellipsoidal shape with a smooth, rigid, hollow, and mostly homogeneous surface lacking porous structure (Fig. 2–D). On the contrary, silica minerals, the main constituent of sea sand, have been found to contain wide pores, which facilitated their utilization as natural adsorbents for enzymes, such as cutinase and xylanase [69, 70]. Whereas [71, 72], found that sand grains absorbed the nutrients poorly with limited ability to form biofilms during their application in biofilters for water purification. Furthermore [73], declared that sand particles displayed the lowest quantity of microbial immobilization owing to their trigonal crystallinity and hexagonal lattice structure. Meanwhile, the smooth hollow sea sand particles appeared aggregated and enclosed throughout the fungal hyphae (Fig. 2–E).

However, PS and LS biowastes showed a clear variation in their microstructure surface morphology despite their lignocellulosic nature. Wherein, PS displayed irregular, loosely stacked flakes or scale-like morphology with some particles containing many fractures and several holes (Fig. 3–A, blue arrows), which was consistent with that obtained by [74]. Interestingly, such a looser surface structure could easily improve the adsorption performance as referred by [62]. Thus, despite low porosity, the fungal hyphae appeared surrounding all the exposed surface area of PS and coated their particles entirely by filling the internal pores (Fig. 3–B). In this context [75], highlighted that porosity and folding, as well as the good bio-affinity of pristine (i.e., without any surface modification) PS, play a crucial role in increasing its surface area and boosting more sites for housing sulfonamide-degradation bacteria. Our results harmonized with those found by [76, 77]. Meanwhile, LS exhibited a polygonal fibrous multi-layered framework with large poly pores in different sizes (Fig. 3–D), representing the entry key for the adsorbate as revealed by [60]. Remarkably, [53], illustrated that the macropores in the fibrous structure of the *Luffa cylindrica* biosorbent contributed to the diffusion of Zn^2+^, Cu^2+^, Pb^2+^, and Ni^2+^. Upon immobilizing by fungal culture, the large cavities appeared blocked, tightly packed, and filled with hyphal biomass (Fig. 3–E). Nonetheless, the interconnected multicellular wires seemed to be devoid of fungal cells. Generally, it could be deduced from SEM micrographs that the biocompatibility of the applied bio-carriers, owing to their natural origin, assisted the higher fungal growth with ease of attachment. Arguably, the firm adhesion between the used bio-carriers and *A. terreus* cells could be attributed to electrostatic forces and covalent interaction between the fungal cells and the applied matrices, which were implemented not only on their surfaces but also within vacuous spaces or matrix pores as denoted by [18].

Finally, while employing the whole hybrid systems of dead fungal-biowaste matrices as biosorbents, a distinct alteration in the surface morphologies was observed. Wherein, *A. terreus* immobilized on SS and CB behaved differently (Fig. 2–C & F). As noticed, bright deposits of Co^2+^ were agglomerated and scattered along the surface of immobilization systems (Fig. 2, red arrows). However, the lignocellulosic residues, namely, LS and PS, showed less regular, smoother, less porous surfaces with more wrinkling and unidentified floccus that seemed to be glued to each other, forming a coat-like structure (Fig. 2-C & F). Seemingly, such a muddy-like appearance characterized *A. terreus* immobilized-sponge biosorbent after heavy metal remediation [45, 78].

### Cobalt biosorption by *A. terreus* immobilized on biowastes

**Fig. 4:**
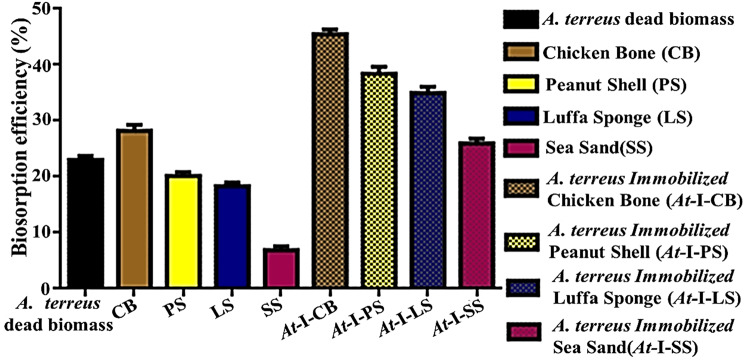
Biosorption performance of *A. terreus* and biowastes in their solitary and immobilized phases

Intrinsically, the current study focused on the development of a different Co^2+^ adsorption medium, composed of *A. terrus* in its dead biomass immobilized on biowaste carriers. Inceptively, it is reasonable to assess the biosorption performance of dead fungal biomass and all examined bio-carriers, in their solitary phase before fungal immobilization, in parallel as well. The examined strain *A. terrus-*OQ275244 possessed the capability to remove 57.5 ± 1.2 mg from a solution of 250 mg L^−1^, recording hereby removal percentage that was calculated by 23.0 ± 0.48%. While the CB displayed the highest capability to adsorb 70.45 ± 1.95 mg L^−1^ of Co^2+^ with biosorption efficiency recorded (28.18 ± 0.78%). Notably, the lowest biosorption capability was shown by SS at 6.9 ± 0.65%, which removed only 17.25 ± 1.62 mg L^−1^ of Co^2+^ from the reaction solution. Besides, a remarkable moderate remediation efficiency was observed by both PS and LS, which remediated 45.75 ± 1.15 and 50.35 ± 1.25 mg L^−1^ of Co^2+^ by recording removal percentages of 20.14 ± 0.5% and 18.3 ± 0.46%, sequentially (Fig. 4).

As manifested by [79], the fungal immobilization on carriers would be advantageous and, in turn, provide additional merits compared to the freely suspended fungal biomass. Thus, the fungal biomass in its solitary phase would execute limited remediation performance against heavy metals or any other pollutant owing to the fewer adsorption sites available. In contrast, the tight fixation of fungal biomass on immobilizing carriers would increase surface area with higher porosity and, subsequently, more vacant adsorption sites for chelating pollutant ions. Collectively, all of these witnesses were clearly evidence in the present study (Fig. 4). Wherein, a synergistic effect or mutualism could describe the adsorptive performance of *A. terreus* immobilized on the used biowastes. Actually, the entire hybrid immobilized fungal-biowaste systems succeeded in remediating Co^2+^ ions more proficiently than each separate partner but in different degrees. A strong uptake property of *A. terreus* immobilized CB (*At*-I-CB) was emphasized through ICP-OES, which assessed the remediated Co^2+^ ions from solution by 113.5 ± 1.65 mg L^−1^ with biosorption efficiency of 45.42 ± 0.66%, representing thereby the highest remediation performance. On the other hand, *A. terreus* immobilized SS (*At*-I-SS) biosorbed only 64.8 ± 2.26 mg L^−1^, which equaled 25.92 ± 0.9%. Intriguingly, approximate biosorptive behavior was demonstrated by both *A. terreus* immobilized on lignocellulosic residues. *A. terreus* immobilized PS (*At*-I-PS) removed about 95.95 ± 2.25 mg L^−1^ by remediation performance reached 38.38 ± 0.9%. Meanwhile, *A. terreus* immobilized LS (*At*-I-LS) adsorbed 87.3 ± 2.0 mg L^−1^ by remediation performance of 34.92 ± 0.8%. Generally, the immobilization process on pristine biowastes enhanced the biosorption of Co^2+^ ions, while the variation in their bioremediation performance could be attributed to their difference in nature, surface area, porosity, adsorptive sites availability, and functional groups variability.

Henceforth, to achieve the most out of the fungal-biowaste hybrid system in scavenging Co^2+^ ions, *At*-I-CB would be selected as a promising unconventional and cost-effective biosorbent, in the subsequent stages of the study. More explicitly, maximizing the biosorption efficiency through statistical modeling followed by comprehending deep kinetics and thermodynamics features of the biosorption process are the main consequent targets.

### Maximizing Co^2+^ biosorption of fungal-immobilized chicken bones via Box-Behnken design

As well-known, biosorption is a complex process controlled by multiple interrelated parameters, including biosorbent dose, concentration of contaminants, and solution chemistry. Evaluating these factors can be achieved by the traditional one-variable-at-a-time (OVAT) approach or mathematical approaches. Arguably, OVAT is expensive and time-consuming because it examines numerous parameters independently. In contrast, numerical techniques such as Response Surface Methodology (RSM) can be utilized to analyze the interactions between variables in both univariate and multivariate models [80, 81]. Among RSM designs, Box-Behnken design (BBD) is one of the multivariate optimization methods based on the second-order response surface model’s three-level fractional factorial designs [82]. Due to lower experimentation costs and more effective experimental design, BBD is the most preferred among other response surface designs, such as three-level complete factorial design, central composite design (CCD), and Doehlert matrix (DM), for the quadratic model, according to [83, 84]. Herein, the impact of pH, *At*-I-CB weight, contact time, and Co^2+^ concentration on the biosorption process was investigated. The interaction between four distinct independent process parameters and the elimination percentage of Co^2+^ ions was investigated in a total of 29 experiments. Table 1 displayed the whole experimental design as well as the actual and expected results, which indicated a high degree of correlation between them.

### Analytical statistics

The significance was assessed at probability levels by the *F* test (*p* ≤ 0.05), while the 3-D surface/2-D contour plots and ANOVA were established for explaining the correlations between the independent variables and response as well as for figuring out the ideal conditions. The determination coefficient (R^2^) was used to estimate the model’s best fit, and the quadratic polynomial equations were created by holding one of the independent variables at a constant value while varying the level of the other variables. The probability values (*P*-value and F-value), which were presented in Table 2, were used to assess each coefficient’s significance.

**Table 2:** ANOVA results for the BBD in determining the percentage of Co^2+^ ions removal by *At*-I-CB hybrid system

Source	Sum of Squares	df	Mean Square	*F*-value	*P*-value	significant
**Model**	20998.99	14	1499.93	465.93	< 0.0001	
${X_1}$(pH)	511.37	1	511.37	158.85	< 0.0001	
${X_2}$(*At*-I-CB Weight)	1193.24	1	1193.24	370.67	< 0.0001	
${X_3}$(Contact Time)	1927.01	1	1927.01	598.60	< 0.0001	
${X_4}$- (Co^2+^ Concentration)	9243.68	1	9243.68	2871.44	< 0.0001	
${X_1}{X_2}$	2.37	1	2.37	0.7362	0.4053	
${X_1}{X_3}$	156.65	1	156.65	48.66	< 0.0001	
${X_1}{X_4}$	557.72	1	557.72	173.25	< 0.0001	
${X_2}{X_3}$	246.34	1	246.34	76.52	< 0.0001	
${X_2}{X_4}$	23.24	1	23.24	7.22	0.0177	
${X_3}{X_4}$	18.75	1	18.75	5.82	0.0301	
$X_1^2$	2551.87	1	2551.87	792.71	< 0.0001	
$X_2^2$	797.52	1	797.52	247.74	< 0.0001	
$X_3^2$	1259.62	1	1259.62	391.29	< 0.0001	
$X_4^2$	1787.89	1	1787.89	555.39	< 0.0001	
Residual	45.07	14	3.22			
Lack of Fit	40.75	10	4.08	3.78	0.1061	n.s.
Pure Error	4.32	4	1.08			
Cor Total	21044.06	28				

*Significant values, *df*: degree of freedom, *F*: Fisher’s function, *P*: level of significance, and n.s.: not significant

The data in Table 3 were fitted to a second-order polynomial equation after the coefficients of the regression equation were computed. The following equation can be used to represent an observed association between removal % and input test variables in coded terms:

14 $$\eqalign{ & C{o^{2 + }}biosorption\,\% \cr & = 55.37 + 6.53{X_1} + {\rm{ }}9.97{X_2} + 12.67{X_3} - 27.75{X_4} + {\rm{ }}0.7697{X_1}{X_2} \cr & + {\rm{ }}6.26{X_1}{X_3} - 11.81{X_1}{X_4} + {\rm{ }}7.85{X_2}{X_3} + 2.41{X_2}{X_4} + 2.16{X_3}{X_4} \cr & - 19.83X_1^2 - 11.09X_2^2 - {\rm{ }}13.94X_3^2 + 16.60X_4^2 \cr} $$

**Table 3:** Regression statistics of the BBD and the coefficients of the second-order polynomial model for the Co^2+^ biosorption by *At-*I-CB

Factor	Coefficient Estimate	Standard Error	95% CI Low	95% CI High
Intercept	55.37	0.8024	53.65	57.09
${X_1}$	6.53	0.5179	5.42	7.64
${X_2}$	9.97	0.5179	8.86	11.08
${X_3}$	12.67	0.5179	11.56	13.78
${X_4}$	−27.75	0.5179	−28.87	−26.64
${X_1}{X_2}$	0.7697	0.8971	−1.15	2.69
${X_1}{X_3}$	6.26	0.8971	4.33	8.18
${X_1}{X_4}$	−11.81	0.8971	−13.73	−9.88
${X_2}{X_3}$	7.85	0.8971	5.92	9.77
${X_2}{X_4}$	2.41	0.8971	0.4863	4.33
${X_3}{X_4}$	2.16	0.8971	0.2408	4.09
$X_1^2$	−19.83	0.7045	−21.35	−18.32
$X_2^2$	−11.09	0.7045	−12.60	−9.58
$X_3^2$	−13.94	0.7045	−15.45	−12.42
$X_4^2$	16.60	0.7045	15.09	18.11
Std. Dev.	1.79	R^2^		0.9979
Mean	43.68	Adjusted R^2^		0.9957
C.V. %	4.11	Predicted R^2^		0.9885
PRESS	241.47	Adeq Precision		69.4134

Coefficient of variation, press: sum of squares of prediction error

In Eq. (14) *X*_*1*_*, X*_*2*_*, X*_*3*_ and *X*_*4*_ are independent singular factors, whereas *X*_*1*_*X*_*2*_, *X*_*1*_*X*_*3,*_ *X*_*1*_*X*_*4,*_ *X*_*2*_*X*_*3,*_ *X*_*2*_*X*_*4*_ and *X*_*3*_*X*_*4*_ are interaction factors and the quadratic terms include *X*_*1*_^*2*^, *X*_*2*_^*2*^, *X*_*3*_^*2*^ and *X*_*1*_^*4*^.

Analysis of variance (ANOVA) for the biosorption of Co^2+^ ions by *At-*I-CB, as an adsorbent, ensured model adequacy and effectiveness. According to Table 2, the model possessed *p* – value less than 0.0001 with F-value fit statistic by 465.93, which indicated that it was significantly accurate and trusted; the coefficient of variation was 15.49%. The adjusted R-squared value (Adj. R^2^ = 0.9957) and the R-squared value (R^2^ = 0.9979) were both near to 1 (Table 3). Remarkably, the model terms are considered significant if their *P*-values were below 0.05. In this instance $$\begin{aligned}{X_1},{X_2},{X_3},{X_4},{X_1}{X_3},{X_1}{X_4},{X_2}{X_3},{X_2}{X_4},{X_3}{X_4}, \cr \quad \quad \quad \quad \quad \quad \quad \quad \quad \quad \quad \quad X_1^2,X_2^2,X_3^2,X_4^2\end{aligned}$$ were constituted the significant model terms (Table 2). The signal to noise ratio was measured using a precision ratio, and the value of 69.413 was an appropriate. This model can therefore be used to explore the design space. Besides, Lack of fit had an *F*-value of 3.78, which reflected its insignificance. There was a 10.61% chance that could occur owing to noise according to the lack of fit *F-*value. When a quadratic model exhibited a negligible lack of fit ( > 0.05), it could be concluded that the model was accurate for the Co^2+^ biosorption by *At-*I-CB. On the other hand, Supplementary Fig. 1 demonstrated how actual and expected values interact. It is evident that the distribution of real values is close to being straight, demonstrating the model’s excellent fitness [85, 86]. Additionally, by creating a normal probability curve of the residuals, it is possible to verify the assumptions of normality (Supplementary Fig. 2). It is plausible to mention that the link between studentized residuals versus expected and the residuals’ random distribution, which was around 3.00, showed that the model was more suited to fit experimental data.

#### Interactive factors

**Fig. 5:**
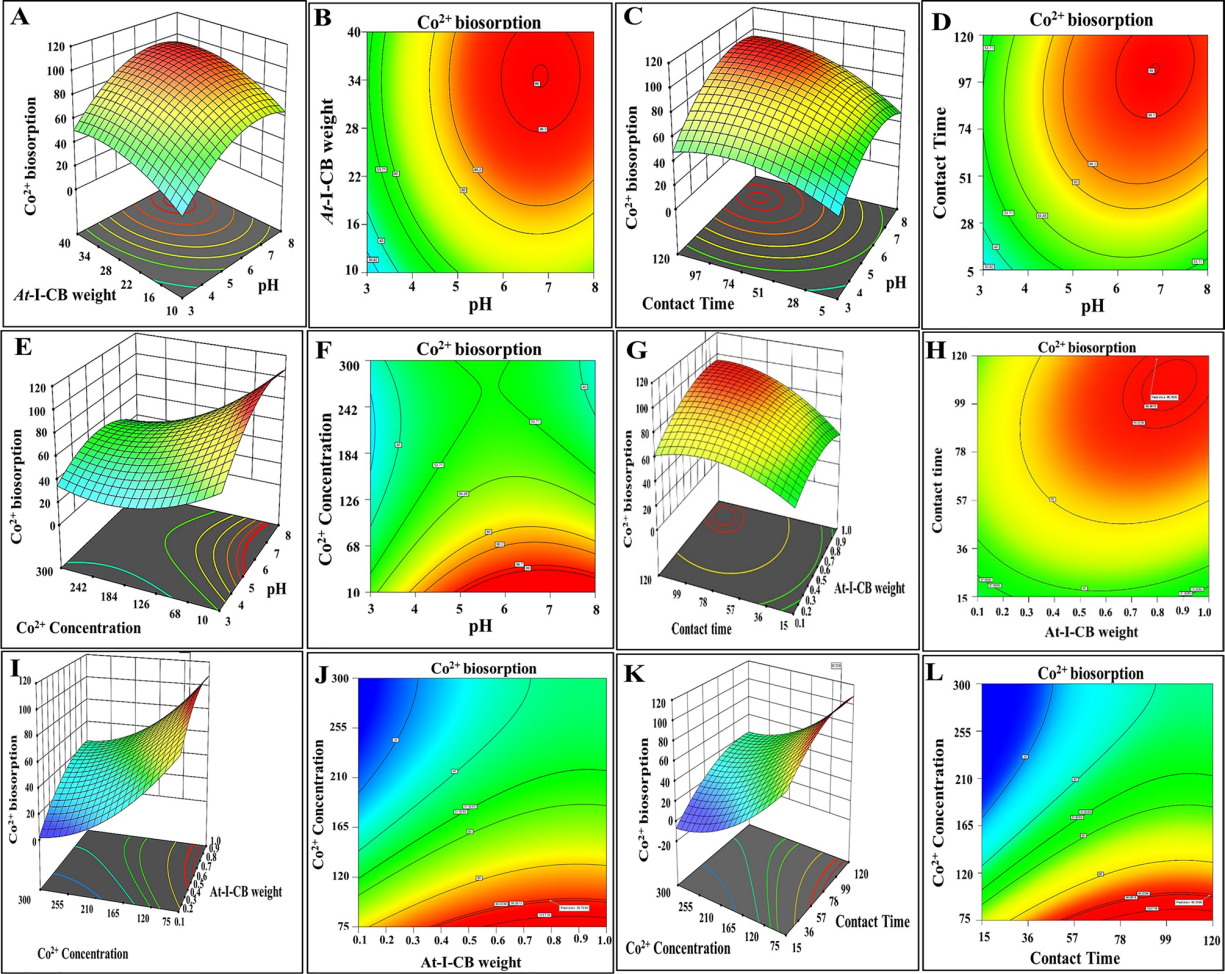
Three-dimensional surface and two-dimensional contour plots for Co^2+^ ions biosorption by *At-*I-CB showing the interactive leverage of the pH, Co^2+^ ions concentration (mg L^−1^), *At-*I-CB weight (g L^−1^) and contact time (min). The plots were constructed by design-expert 13

The experimental strategy used four process variables and a hierarchical quadratic model to describe graphically the perfect conditions for Co^2+^ biosorption by *At-*I-CB via 3D and 2D plots. Graphical representations known as 3-D surfaces were created by plotting the response (Co^2+^ biosorption %) on the Z-axis against two independent factors while keeping the other factors at their zero level, in order to identify the ideal conditions for biosorption of cobalt. While 2D-contour plots were used to find their interactions (Fig. 5). In addition, such graphical plots were used to explain the relationship between each pairwise combination of the variables (${X_1}{X_2}$,${X_1}{X_3}$,${X_1}{X_4}$, ${X_2}{X_3}$,${X_2}{X_4}$, and ${X_3}{X_4}$) and the responses. Elliptical contour plots reveal strong significant interactions between factors, whereas circular contour plots point out to insignificant interactions. Surface constrained in the smallest ellipse of contour diagrams revealed the maximum expected yield, a considerable interaction effect on the rate of adsorption [87]. The optimum values are obtained from the best 100 random runs using the numerical optimization technique in Design Expert software.

## Impacts of pH and *At*-I-CB weight

Figures 5-A and B displayed a three-dimensional surface and the associated contour plot demonstrating the interactive effect of pH and *At*-I-CB weight on Co^2+^ ion removal. Surface constrained in the smallest circular of contour diagram showed the maximum expected yield, and both variables had a synergistic interaction. At pH 3.1 and *At*-I-CB weight (1.1 g L^−1^), Co^2+^ removal efficiency was 28.4%, and it increased to 95.7% at pH 6.76 and *At*-I-CB weight (8.7 g L^−1^), indicating a potent interaction between metal cation and the negatively charged binding sites on the biosorbent at nearly neutral pH. The biosorbent’s capacity to bind Co^2+^ ions rose with increasing initial pH up to 6.9 but thereafter decreased at alkaline pH. The lower biosorption at higher pH (i.e., ˃ 7) could be most likely owing to the precipitation of Co^2+^as Co(OH)_2_. Similarly, the absorption of Co^2+^ increased with an increasing pH from 4.5 to 7 in *Paecilomyces catenlannulatus* [88]. As the pH rises, more ligands appear, and the number of negatively charged groups on the adsorbent matrix increases, enhancing biosorption of all cationic species [89, 90]. Compared with chemically modified composites, which had the greatest adsorption of Lu^3+^ and Cd^2+^ at acidic pH levels (4.0 and 3.5, respectively) [91, 92], Co^2+^adsorption onto our bio-based adsorbent was weak at acidic pH levels and the highest value at a neutral pH level (6.76). This implied how protonation states and the type of adsorbent affect how much metal is absorbed [91, 92]. The efficacy of the biosorbent in removing Co^2+^ ions increased with rising *At-*I-CB weight to 8.7 g L^−1^. This finding agreed with the research conducted by [93] who demonstrated that chicken bone ash (CBA) exhibited significant effectiveness in phenol adsorption due to its plentiful surface-active sites. Their investigation further established that adsorption efficiency was markedly affected by pH, with optimal removal occurring at neutral value. Conversely, elevating the pH to 9 resulted in a decrease in adsorption capacity to 73.6%, attributed to diminished electrostatic attraction between phenol molecules and the negatively charged CBA surface.

## Impacts of pH and contact time

Figures 5-C and D showed a 3D surface and its related contour plot, illustrating how pH and time of contact collaborated significantly to remove Co^2+^ ions. Wherein, Co^2+^ ions removal percentage was uplifted with elevating contact time and pH; these findings were consistent with previous studies [94, 95]. More specifically, at acidic conditions (pH 2.72) and a short contact period (28.36 minutes), the removal effectiveness was just 29.32%. However, at near-neutral pH (6.66) and a longer contact duration (102.85 minutes), efficiency increased dramatically to 95.7%. This improvement can be attributed to two important elements. At low pH, H^+^ ions compete with Co^2+^ ions for active sites on the biosorbent surface, which subsequently reduce Co^2+^ uptake [96]. As pH rises, competition lessens, leading to improved ion exchange and electrostatic interactions between Co^2 +^ and negatively charged functional groups on the biosorbent. Meanwhile, a prolonged contact period permitted more Co^2+^ions to interact with accessible binding sites, leading to gradual surface saturation and better removal before equilibrium [97] Likewise [91, 92], declared that optimizing contact time would in turn boosted the elimination of Lu^3+^ and Cd^2+^by ligand-based composite hybrid nanomaterials (CMHs) and optical composite materials (OCM), correspondingly.

## Impacts of pH and cobalt concentration

In Fig. 5-E and F, 3D surface and 2D plots elucidated the significant antagonistic impact of pH and initial cobalt concentration on Co^2+^ biosorption. Herein, the optimum contour ranges for Co^2+^ content (75.83 - 104.6 mg L^−1^) and pH (4.79 - 8.1) yielded a maximum removal effectiveness of 95.7%. The plots revealed that Co^2+^ removal effectiveness initially rose at lower ion concentrations but dropped as concentrations increased. This decline is most likely caused by the biosorbent’s limited number of active adsorption sites, which become saturated when the initial ion concentration surpasses a specific threshold [90, 98]. Notably, the findings of [87, 95] were harmonized with our obtained results.

## Impacts of *At*-I-CB weight and contact time

Figures 5-G and H depicted a 3D and 2D plot that illustrated the significant interactive effects of *At-*I-CB weight and contact time on the elimination of Co^2+^ ions. While all other variables were maintained at their optimal values. Synergistically, increasing the values of both parameters resulted in higher removal efficiency, reaching a maximum of 95.8% within a contact time range of 100.37–106.57 minutes and an *At*-I-CB weight between 8.05 and 8.56 g L^−1^. Prior studies displayed that the amount of adsorbent/catalyst influenced on adsorption efficiency through active site availability [2, 92]. The observed increase in removal rate could be explained by the availability of unoccupied active sites on the biosorbent surface during the early phase. However, prolonging contact duration beyond this range caused saturation of the biosorbent surface, resulting in equilibrium with no further substantial adsorption. These findings were consistent with prior scholars [99, 100], which reported that the removal efficiency of Ni (II) ions and methylene blue by utilizing G*racilaria* improved with contact time and peaked at 177.88 minutes, emphasizing the importance of contact time optimization in biosorption processes.

## Impacts of *At*-I-CB weight and cobalt ion concentration

Figures 5-I and J demonstrated the 3D and contour plots of the significant interaction exerted by *At*-I-CB weight and initial Co^2+^ concentration on its elimination under optimal conditions. At a low *At-I*-CB weight (3 g L^−1^) and a high cobalt concentration (241 mg L^−1^), the removal efficiency was just 36%. In comparison, at a higher adsorbent dose (8.59 g L^−1^) and a lower Co^2+^ concentration (102.62 mg L^−1^), the removal effectiveness reached 95.7%. As such, it is conceivable that higher concentrations of sorbate results in diminishing the absorption efficiency due to competition for limited binding on sites biosorbent’s surface [88]. Conversely, raising the adsorbent dosage improved removal efficiency, most likely due to increased surface area and number of accessible active sites. Nonetheless, at greater concentrations of biosorbent’s, adsorption tends to stabilize when the sorbate-sorbent ratio becomes imbalanced, resulting in a reduction in contact surface area per unit mass of sorbent. This behavior is similar to trends reported in other research using bone-based adsorbents. In comparison, earlier research on metal ion adsorption found that initial metal concentrations enhanced absorption rapidly until the active sites of the adsorbent were saturated by increasing the driving force for mass transfer. In the same context [92], and [88] pointed out to the leverage of the initial metal ion concentration on the whole sorption process, underscoring the significance of surface interactions and active site availability.

## Impact of cobalt ion concentration and contact time

Figures 5-K and L exhibited the surface and contour plots of the significant interaction between contact time and initial Co^2+^ concentration on biosorption efficacy. At a cobalt concentration of 212 mg L^−1^, which was high, and a short contact duration of 42.3 minutes, the elimination efficiency was only 36%. The decreased performance at high metal concentrations and short exposure times could be explained by the saturation of accessible active sites on the biosorbent surface, where the high ionic load exceeds the adsorption capacity during early phases of contact. In contrast, extending the contact time to 63 minutes with lowering the initial Co^2+^ concentration to 71 mg L^−1^ resulted in 80% removal efficiency. According to contour analysis, the best range was observed at Co^2+^ concentrations of 75 to 110 mg L^−1^ within contact time of 50 - 109 minutes, which achieved a maximum removal of 95.66%. This pattern was consistent with the data published by [101], who found that high concentrations of heavy metal may impede adsorption due to competition for limited binding sites. Similarly, studies conducted by [102, 103], suggest that adequate contact time is necessary to attain adsorption equilibrium, especially when biosorbent surfaces require progressive interaction with metal ions for enhanced absorption efficacy. In addition, it was highlighted that prolonged contact duration enhances the potential of metal ions interacting with active functional groups; however, high initial metal concentrations can decrease removal effectiveness due to quick saturation of accessible sites [104].

## The model’s validation

According to second-order polynomial models, the *At*-I-CB hybrid system fulfilled the highest cobalt removal from aqueous solution under the optimized conditions, which included cobalt concentration of 103.04 mg L^−1^, *At*-I-CB weight 8.7 g L^−1^ and pH 6.76, within contact time of 101.4 min, as shown in the perturbation graph (Supplementary Fig. 3). Under these conditions, the maximum cobalt removal percentage of 96.8% was confirmed and compared to the value anticipated by the polynomial model (95.7%). Validation revealed high model accuracy, indicating the model’s validity at the concentrations used.

## Confirming Co^2+^ biosorption on *At*-I-CB under optimized conditions

**Fig. 6:**
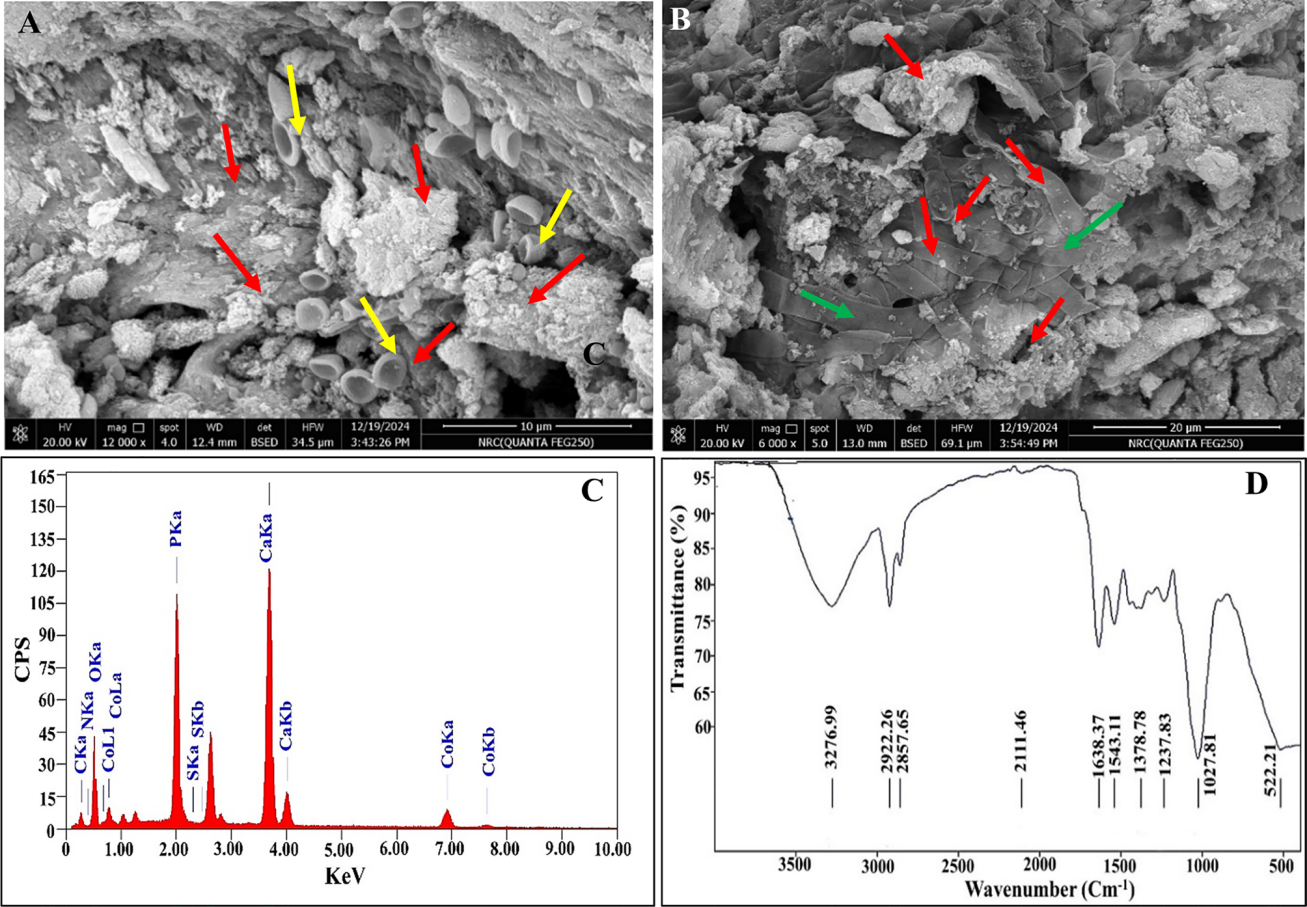
Characteristic properties of *At-*I-CB hybrid system after treatment of Co^2+^ under optimized conditions predicted from BBD confirming the achievement of biosorption. A)-SEM field depicted conidia immobilized CB with adsorbed Co^2+^, B)- SEM field depicted hyphae immobilized CB with adsorbed Co^2+^. C)-EDX pattern and D)- FTIR spectral profile. Red arrows, adsorbed Co^2+^, green arrows: fungal hyphae and yellow arrows: fungal conidia

Eventually, the *At-*I-CB hybrid system was examined under optimized conditions to emphasize the biosorption efficiency through other complementary analytical techniques. In this sense, the surface topography of the *At-*I-CB hybrid system after treatment was accentuated by SEM (Fig. 6-A and -B). Apparently, the adsorbed hybrid system, under optimum conditions, detected higher amounts of Co^2+^ ions than that detected in the initial screening process (Fig. 3–C). That could be evident through the presence of a higher distribution of shiny particles not only over the surfaces of fungal conidia and hyphae but also over the surface of the bio-carrier, reflecting the efficacy of the optimization process in maximizing the adsorptive properties of the whole system of fungal-based biowaste. In this sense, as stated by [105], the adsorption process was delineated as a surface phenomenon, where its efficiency based mainly on several factors, including the abundance of active sites, large pore size, and high surface area. On the other hand, the elemental microanalysis using EDX unveiled the elemental profile that constituted the treated samples. Broadly, the examined profile affirmed the hydroxyapatite framework of the immobilization matrix through the presence of remarkable signals at 0.277, 0.52, 2.01, and 3.69 keV with atomic percentages of 11.56, 57.52, 11.29, and 14.29 % that identified for carbon, oxygen, phosphorous and calcium, respectively. However, an additional signal for nitrogen was also observed at a binding energy of 0.392 keV with a percentage of 3.07% (Fig. 6–C), which could be fungal origin attributed to proteinaceous components of fungal cells. The signal of cobalt was noticed at 6.92 keV with an atomic percentage of 2.26%, which indicated the integration of cobalt into the biosorbent composite. It is plausible to mention that the involvement of carbon and oxygen as intrinsic elements in the remediated sample was deemed as an appreciable key in the whole Co^2+^ adsorption process. Such conclusion could be ascribed to the covalent bond that formed between Co^2+^ ions and the functional groups, which are disseminated on the surface of the biosorbent hybrid system. Both elements are considered the fundamental elements of various functional groups such as C=O, O-H, etc. [105], as would be declared later on by FTIR.

However, understanding the biosorption mechanism and perceiving the probable complexation or sequestration of biosorbent–adsorbate ions necessitate defining their surface functional groups. The FTIR spectrum of *At-*I-CB treated with Co^2+^, in wavelength ranges from 500 cm^−1^ to 3500 cm^−1^, was given in Fig. 6–D. Initiating by the FTIR peak around 3276 cm^−1^, it could be attributed to O-H and N-H stretching vibration groups, according to the findings of [106]. Meanwhile, the absorbance band located at 2922 cm^−1^ was correlated with alkyl and CHO. This range of vibration was discovered in *A. terreus*, and this finding was consistent with [107]. The bands at 1638 cm^−1^ and 1543 cm^−1^ could be assigned to the bending vibration of the deformation bands C=O (carbonyl) and N-H (amide), respectively, as highlighted by [108]. Besides, the absorbance band at 1543.11 cm^−1^ corresponds to amide II, which is involved in the motion incorporating both the NH bending and the CN stretching vibration of group C(O)-NH in its transformation [109]. While the waveband at 1378 cm^−1^ was ascribed to amide bands (amide III) [110]. Notably, the C-O asymmetric C-O-C stretching ester vibration could be implied from the peak at 1237 cm^−1^ [111]. Strikingly, a prominent peak around 1027 cm^−1^ was detected, corresponding to phosphate (PO₄) bonds, which is a hallmark of hydroxyapatite (Ca₁₀(PO₄)₆(OH)₂) that constitutes the bones structure [112, 113]. This peak also could unveil the presence of C-O-C and  P=O stretching vibrations within the fungal components [114]. A noticeable major peak was detected at 522 cm^−1^, which reflected the presence of cobalt oxide bonds within the structure of the hybrid biosorbent system. As revealed by [115], the spectra peaks at 568, 576, 670, and 662 cm^−1^ characterize the stretching vibration mode of Co-O. Besides [116, 117], referred that the FTIR fingerprint region below 1000 cm^−1^, particularly the range of 400–700 cm^−1^, identified the metal-oxide bond, which was in sync with our results. Generally, these spectral features confirmed the successful integration of Co^2+^ with hydroxyapatite-fungal components in the composite material. It is well grasped that the hydroxyapatite matrix played an important multifaceted role in cobalt biosorption. Wherein, it provides structural support for the fungal biomass, enhancing its stability and potentially improving its accessibility to metal ions. In addition, hydroxyapatite itself possesses phosphate groups that can interact with cobalt ions, contributing to the overall biosorption capacity. The presence of calcium in hydroxyapatite can also influence the surface charge and potentially facilitated metal binding. Moreover, hydroxyapatite may act as a nucleation site for metal precipitation, which further enhanced cobalt removal [112, 113, 118]. Let alone the vigor adsorptive role of fungal macromolecules including polysaccharides, structural proteins, and lipids in the fungal cell wall that supply with many functional groups for metals attachment (e.g., carboxylate, hydroxyl, phosphate, amine, and sulfate) [119]. Finally, previous scholars revealed the presence of functional ionizable groups, and their ionization leaves empty sites that metals can fill [110].

### Effect of the contact time on the adsorption of Co^2+^ by *At*-I-CB hybrid system

**Fig. 7:**
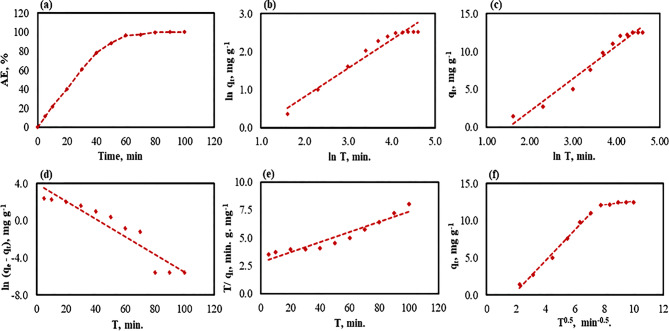
Effect of contact time on the adsorption of cobalt (Co^2+^, 100 mg L^−1^) by *At*-I-CB hydride system; (**a**) adsorption efficiency percentage (AE, %), (**b**) fractional power, (**c**) Elovich, (**d**) pseudo-first order, (**e**) pseudo-second order, and (**f**) intra-particle diffusion

The impact of contact times extending to 100 minutes on the adsorption efficiency (AE, %) of Co^2+^ was carried out under optimized conditions: an initial concentration of 100 mg L^−1^, an adsorbent dosage of 8.7 g L^−1^, and a pH of 6.7 (Fig. 7–a) The results demonstrated that the AE (%) had a sharp increase with continued experimental time in the first stage, then reached a plateau stage, which suggested that the adsorption reached an equilibrium state. Notably, the maximum adsorption capacity (q_e, exp._) reached 12.50 mg g^−1^, realizing an adsorption efficiency of 100% after 80 minutes. Beyond this stage, no progress in the adsorption system was observed, meaning that the equilibrium had been achieved in the Co^2+^/*At*-I-CB system. This could be attributed to saturation of the activated adsorption sites on the *At*-I-CB surface, indicating that all available binding sites were occupied by Co^2+^ ions [120–122].

### Adsorption kinetics of Co^2+^ ions

Adsorption kinetics models clarify the mechanisms governing the adsorption process (i.e., diffusion, mass transfer, and chemical interaction) by describing the mass transfer and reaction rates that occur between the adsorbate molecules and the adsorbent surface over time, from the initial stage of the reaction to the equilibrium state [122, 123]. Several kinetic models were applied to understand the adsorption mechanism of Co^2+^ on *At*-I-CB, including the Fractional power, Elovich model, Pseudo-First Order, Pseudo-Second Order, and Intra-Particle Diffusion. The Fractional power model, derived from the Freundlich isotherm, describes the adsorption process on heterogeneous surfaces, while the Elovich model is commonly used to explain chemisorption processes that follow the second-order kinetics of such surfaces. The Pseudo-First Order assumes that the number of adsorbate molecules matches the number of available active sites on the sorbent material. Meanwhile, the Pseudo-Second Order model relies on the sharing and exchanging of electrons between pollutants and the adsorbent surface, indicating chemical adsorption. The Intra-Particle Diffusion model effectively describes the multi-stage dynamics of adsorption, which includes both external surface interaction and internal pore diffusion of the adsorbate [120–122]. The suitability of the mentioned models for describing Co^2+^ adsorption into *At*-I-CB was evaluated by the correlation coefficient value (R^2^, ranging from 0.0 to 1.0), which reflects its accuracy in illustrating the adsorption mechanism. Based on the data presented in Table 4 and Fig. 7 (b-f), the most fitted models were Intra-Particle Diffusion model (first stage, R^2^ = 0.99), followed by the Fractional Power and Elovich models (R^2^ = 0.96). Both Pseudo-first Order and Pseudo-Second Order models provide a less adequate description of the Co^2+^ adsorption on the surface of suggested sorbent material compared to other tested models. This is evidenced not only by their relatively lower R^2^ values but—more importantly—by the substantial overestimation of calculated adsorption capacity at equilibrium (*q*_*e, cal.*_), which significantly exceeded the experimental value (*q*_*e, exp.*_). These discrepancies suggest that the adsorption process is better represented by a multi-stage mechanism. Wherein, intra-particle diffusion played a key role in the initial rate-limiting step, as supported by the higher R^2^ and more physically consistent parameters of the intra-particle diffusion model.

**Table 4:** Fitting parameters of the common five kinetic model for Co^2+^ adsorption onto *At*-I-CB

Kinetic Model	Parameter	Value
Experimental adsorption capacity	*q*_*e*, *exp*_, mg g^−1^	12.50
Fractional Power	*a*, mg g^−1^	0.50
	*b*, min^−1^	0.75
	*R*^*2*^	0.96
Elovich	*α,* mg g^−1^ min^−1^	0.05
	*β,* g mg^−1^	4.28
	*R*^*2*^	0.96
Pseudo-First Order	*K*_*1*_ min^−1^	−0.1
	*q*_*e, calc.*_, mg g^−1^	52.66
	*R*^*2*^	0.89
Pseudo-Second Order	*K*_*2*_, g mg^−1^ min^−1^	
	*q*_*e, calc.*_, mg g^−1^	22.22
	*R*^*2*^	0.91
Intra-particle Diffusion	*L*_*1*_, mg g^−1^	−3.65
	*D*_*1,*_ mg min^−0.5^ g^−1^	2.07
	*R*^*2*^	0.99
	*L*_*2*_, mg g^−1^	10.49
	*D*_*2,*_ mg min^−0.5^ g^−1^	0.21
	*R*^*2*^	0.82

Generally, the parameters of the Elovich model demonstrate that the adsorption process occurs rapidly, indicating low activation energy and reflecting that the adsorption mechanism is chemisorption through several techniques, such as electrostatic interaction, ion exchange, and complexation [123]. Furthermore, the Fractional Power constant (*b* = 0.75 min^−1^), being less than one, provides that the adsorption mechanism involves both external diffusion on the *At*-I-CB surface and internal pore diffusion. Thus, the Intra-Particle Diffusion model was tested to investigate the impact of Co^2+^ mass transfer on *At*-I-CB [123]. However, the process has two stages depending on the rate of adsorption. The fast stage was realized by the transfer of Co^2+^ from the solution to the *At*-I-CB surface via film diffusion. While the second stage reflected that the entire reaction’s rate through pores was not a limiting step. This was confirmed by the value rate constant of *D*_*s*_. The higher the *D*_*s*_, the stronger the limiting step for the adsorption process (Table 4). Additionally, the *L*_*s*_ value related to the intercept line, which refers to the boundary layer thickness, was significantly greater in the second stage (*L*_*2*_, 10.49 mg g^−1^) than in the first stage (*L*_*1*_, −3.65 mg g^−1^), which reflected that the intra-particle diffusion of Co^2+^ had occurred. From another point of view, the intercept line did not pass through the zero point, meaning the intra-particle diffusion is not the only controlling mechanism of the adsorption process but also the boundary layer diffusion may influence [93, 123]. The obtained results emphasized that the well-developed internal pore structure materials provided reactive adsorption via both external film diffusion and intraparticle diffusion (pore or surface diffusion) [124].

### The temperature and thermodynamic effects of Co^2+^ adsorption onto *At*-I-CB hydride system

**Fig. 8:**
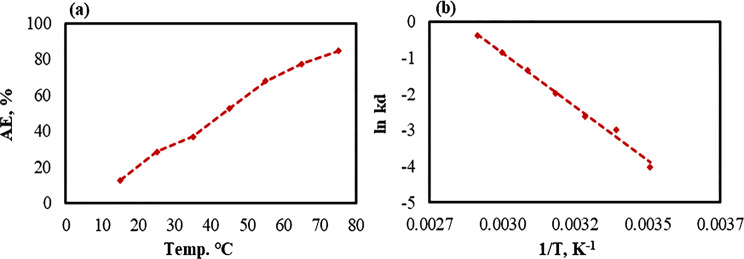
Effect of temperature and thermodynamic on the adsorption of Co^2+^, 100 mg L^−1^ by *At*-I-CB (**a**) adsorption efficiency (AE, %); (**b**) Van’t Hoff plot

The effect of temperature on the adsorption of Co^2+^ ions by *At*-I-CB was investigated over a range of 15 °C to 75 °C to determine the maximum adsorption capacity. The results indicated that adsorption efficiency increased with rising temperature, reaching a maximum of 84.60% at 75 °C (Fig. 8). This improvement in adsorption performance can be attributed to the increased kinetic energy of Co^2+^ ions at higher temperatures, which promoted their mobility and diffusivity, thereby, facilitating more effective interaction with active sites on the *At*-I-CB surface. These observations suggested that the adsorption process is endothermic. Additionally, the increase in temperature may activate the adsorbent surface, further enhancing the adsorption capacity of *At*-I-CB for Co^2+^ ions [125, 126]. To confirm the phenomenon, thermodynamic studies were conducted on the adsorption of Co^2+^ onto *At*-I-CB. Notably, thermodynamic parameters such as ΔH°, ΔS°, and ΔG° are essential for assessing the spontaneity and feasibility of the adsorption mechanism [49, 127]. The negative value of Gibbs free energy (ΔG°) endorses a spontaneous process; whereas, the positive value reflects a non-spontaneous process [127]. (Table 5) showed that the ΔG° values were positive at various investigated temperatures and decreased with increasing temperature, indicating a more energetically favorable process at higher temperatures, and the adsorption of Co^2+^ ions onto the *At*-I-CB is a non-spontaneous process. Furthermore, enthalpy change (∆H°) delivers information related to the nature and mechanism of adsorption processes. The negative value of this parameter indicates an exothermic adsorption process, whereas the positive value reflects an endothermic process [127]. Additionally, the value of ΔH° provides information related to the nature of the adsorption type, physical or chemical. Generally, physical adsorption is associated with a ΔH° value in the range of 2.1–20.9 kJ/mol, whereas chemical adsorption typically occurs in the range of 20.9 and 418.4 kJ/mol at higher energy [128]. In this study, the positive value of ΔH° (49.54 kJ/mol) indicated the endothermic nature of the Co^2+^ ions adsorption process, meaning the heat was absorbed during the process, as well as the adsorption mechanism was chemisorption. The distribution coefficient (K_d_) increased with temperature, from 0.018 L/g at 289 K to 0.688 L/g at 349 K, which indicated the enhancement of adsorption capacity at higher temperatures. Entropy Change (∆S°) is related to the affinity between the adsorbate and the adsorbent surface. The negative value of ∆S° (−139.00 J/mol K) indicated the adsorption of Co^2+^ decreased randomness at the solid-liquid interface during the adsorption process, accompanied by structural changes in both the adsorbent and adsorbate [125, 126].

**Table 5:** Thermodynamic parameters of Co^2+^ adsorption on *At*-I-CB hybrid system

Temperature (K)	**K**_**d**_ **(L/g)**	ΔG° (kJ/mol)	ΔH° (kJ/mol)	ΔS° (J/mol K)
289.15	0.018	9.35	49.54	−139.00
299.15	0.050	7.96		
309.15	0.073	6.57		
319.15	0.138	5.18		
329.15	0.262	3.79		
339.15	0.426	2.40		
349.15	0.688	1.01		

**Fig. 9:**
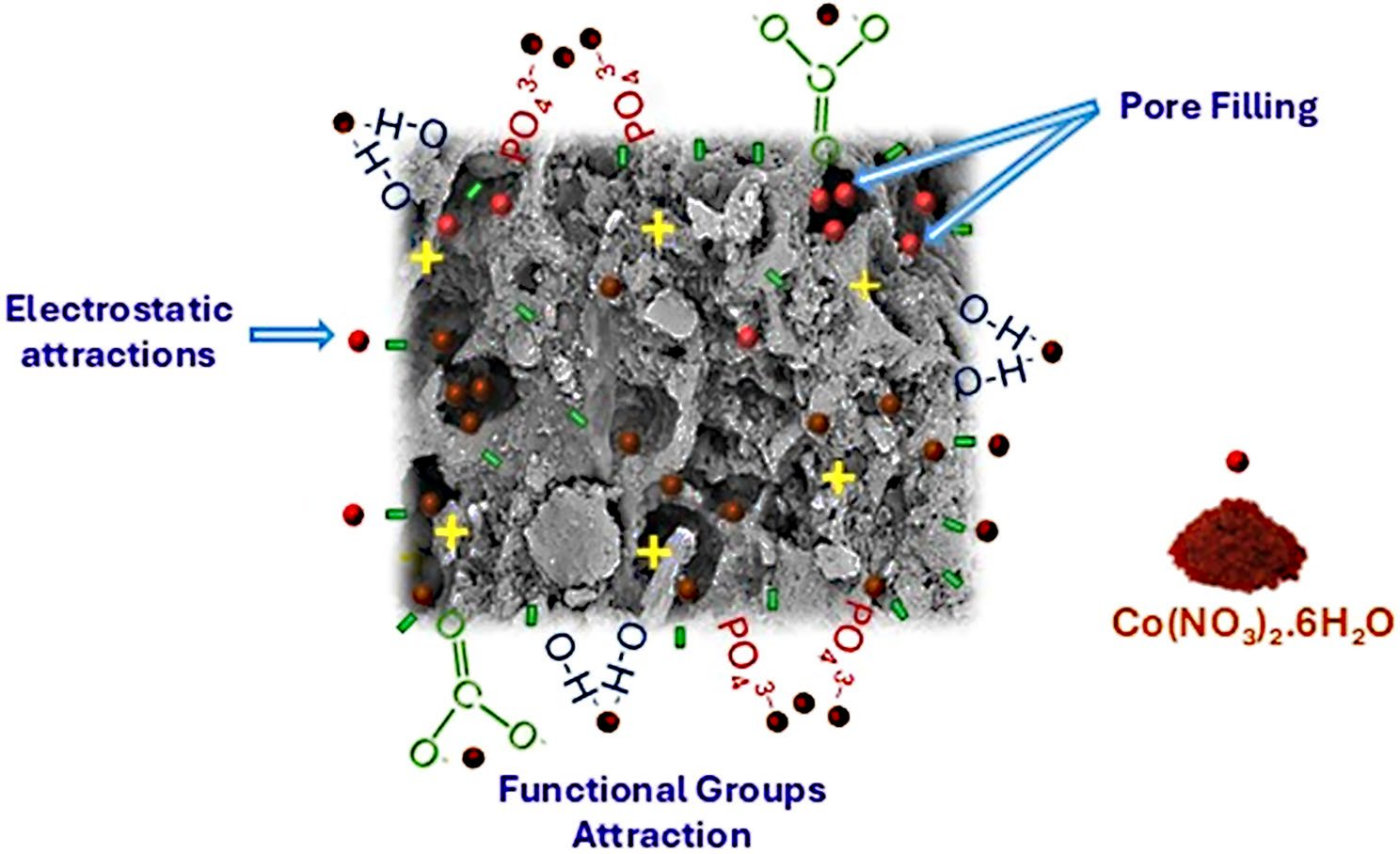
Schematic representation of adsorption mechanism of Co^2+^ onto *At*-I-CB hybrid system

Based on all prior data, the mechanism of remediating CO^2+^ by *At*-I-CB hybrid system could be envisage. Strikingly, several adsorption mechanisms were proposed to interpret the interactions between cobalt ions and fungi-immobilized chicken bones, including electrostatic attraction, ion exchange, complexation, covalent bonding, precipitation, pore filling, chemisorption, and physisorption [120, 122]. The FT-IR study of *At-*I-CB hybrid system presented different functional groups like CO_3_^2-^, OH^-^ and PO_4_^3−^, which furnished negative charges on the surface of *At-*I-CB, thereafter, reacted with positively charged Co^2+^ through a strong electrostatic attraction mechanism. Thus, cationic Co^2+^ ions can easily adhere to the negatively charged *At-*I-CB hybrid surface. Meanwhile, the values of correlation coefficients related to kinetics and thermodynamic studies denoted the predominance of chemisorption mechanism. Nevertheless, the well-developed internal pore structure of *At-*I-CB hybrid implied the accomplishment of adsorption process via both external film diffusion on the adsorbent surface and intraparticle diffusion (pore or surface diffusion). Figure 9 demonstrated the schematic representation of the proposed Co^2+^ adsorption mechanism by *At-*I-CB hybrid system.

## Conclusion

This study successfully examined the efficiency of novel hybrid biosorbent system, comprising dead biomass *of A. terreus* immobilized on chicken bones (*At*-I-CB), for remediating cobalt. Thereby, achieving a dual-purpose approach of heavy metal mycoremediation concurrently with waste management. Among different immobilization matrices (i.e., lignocellulosic, siliceous, and hydroxyapatite-based biowastes), *At*-I-CB exhibited the highest Co^2+^ biosorption capability by 45.42 ± 0.66%. Subsequent optimization using a Box-Behnken design predicted the maximum Co^2+^ removal (96.8%) of initial 103.04 mg L^−1^ of Co^2+^ by 8.7 g L^−1^ of *At*-I-CB at pH 6.76 within 101.4 min. To affirm the success of Co^2+^ biosorption, SEM, EDX, and FTIR were employed, which unveiled the heterogeneous and porous structure of the novel *At*-I-CB hybrid system, integration of cobalt signal throughout hydroxyapatite-fungal microelement, and functional groups that mediated cobalt chelation and complexation. However, kinetic modeling indicated that Intra-Particle Diffusion best described the initial stage of biosorption (R^2^ = 0.99), followed by Fractional Power and Elovich models. This indicated a combined surface interaction, pore diffusion, and boundary layer effects, which recapped the chemisorption tactic of *At*-I-CB hybrid system to biosorb Co^2+^. Furthermore, thermodynamic analysis showed that the adsorption capacity increased with temperature, consistent with an endothermic process (ΔH° = 49.54 kJ/mol). Meanwhile, the positive ΔG° values confirmed a non-spontaneous adsorption. Remarkably, this is the first study to investigate Co^2+^ remediation along with deeply detailed kinetics and thermodynamic modeling of the novel economically viable *At*-I-CB hybrid system. Thus, complying with sustainability goals and techno-economic productivity. Finally, the current study represents the mainstay of a series of future studies concerning remediating wastewater from other heavy metals, antibiotics, pesticides, and azo dyes in realistic multi-component systems.

## supplementary material

Below is the link to the electronic supplementary material.

Supplementary Material 1

## Data Availability

All data generated or analyzed during this study are included in this published article. The 28S-rRNA sequencing technique was performed, and the data was deposited in the GenBank database under the accession number of OQ275244 (https://www.ncbi.nlm.nih.gov/nuccore/OQ275244.1/).
