# Supplementary material for: Strategic utilization of chicken bones immobilized by Aspergillus terreus for sustainable bioremediation of cobalt: statistical modeling, kinetics and thermodynamic studies
Source: J Biol Eng. 2026 Mar 11;20:62. doi: 10.1186/s13036-025-00599-5 (PMC13063698; doi:10.1186/s13036-025-00599-5)
Supplement: Supplementary file 1 — Supplementary Material 1 [file 13036_2025_599_MOESM1_ESM.docx]

**Strategic utilization of chicken bone immobilized by *Aspergillus terreus* for sustainable bioremediation of cobalt: statistical modeling, kinetics and thermodynamic studies**

**Marwa Eltarahony ^1*^, Eman El‑Gamal ^2^,** **Moustafa M Salama ^3,4^, Amany Ibrahim ^5, 6,7^**

^1^ Environmental Biotechnology Department, Genetic Engineering and Biotechnology Research Institute (GEBRI), City of Scientific Research and Technological Applications (SRTA-City),

New Borg El‑Arab 21934, Alexandria, Egypt.

^2^ Land and Water Technologies Department, Arid Lands Cultivation Research Institute (ALCRI), City of Scientific Research and Technological Applications (SRTA-City), New Borg El‑Arab City 21934, Alexandria, Egypt.

^3^ Mathematics Department, Faculty of Science, Taif University, Taif, Saudi Arabia.

^4^ Department of Computer-Based Engineering Applications, Informatics Research Institute, City of Scientific Research and Technology Applications, Egypt.

^5^ Botany Department, Faculty of Women for Arts, Science and Education, Ain Shams University,

Cairo, Egypt.

^6^ Department of Biology, College of Science, Taif University, P.O. Box 11099, 21944 Taif, Saudi Arabia.

^7^ High Altitude Research Center, Taif University, P.O. Box 11099, Taif 21944, Saudi Arabia

*** Corresponding author:**

**Marwa Eltarahony**

E-mail: [m_eltarahony@yahoo.com & meltarahony@srtacity.sci.eg](mailto:m_eltarahony@yahoo.com%20&%20meltarahony@srtacity.sci.eg)

ORCID ID 0000-0002-2739-6231

**Supplementary Figure 1** Relationship between expected and actual values.


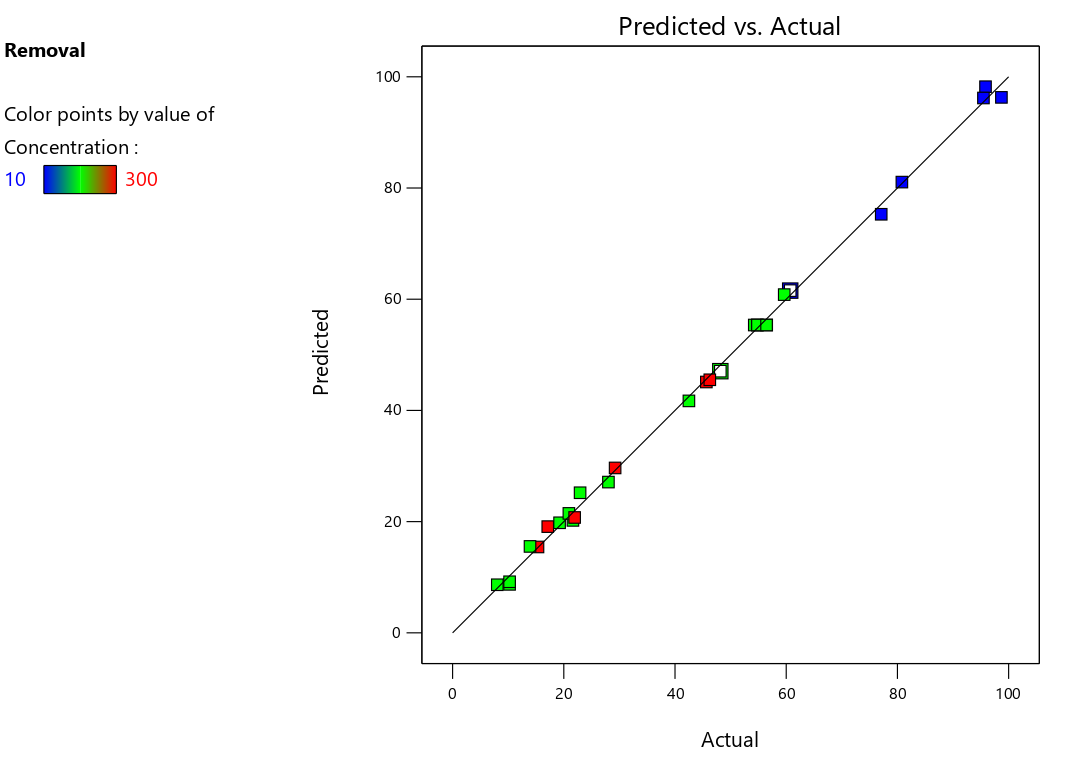


**Supplementary Figure 2** Residues of Externally Studentized


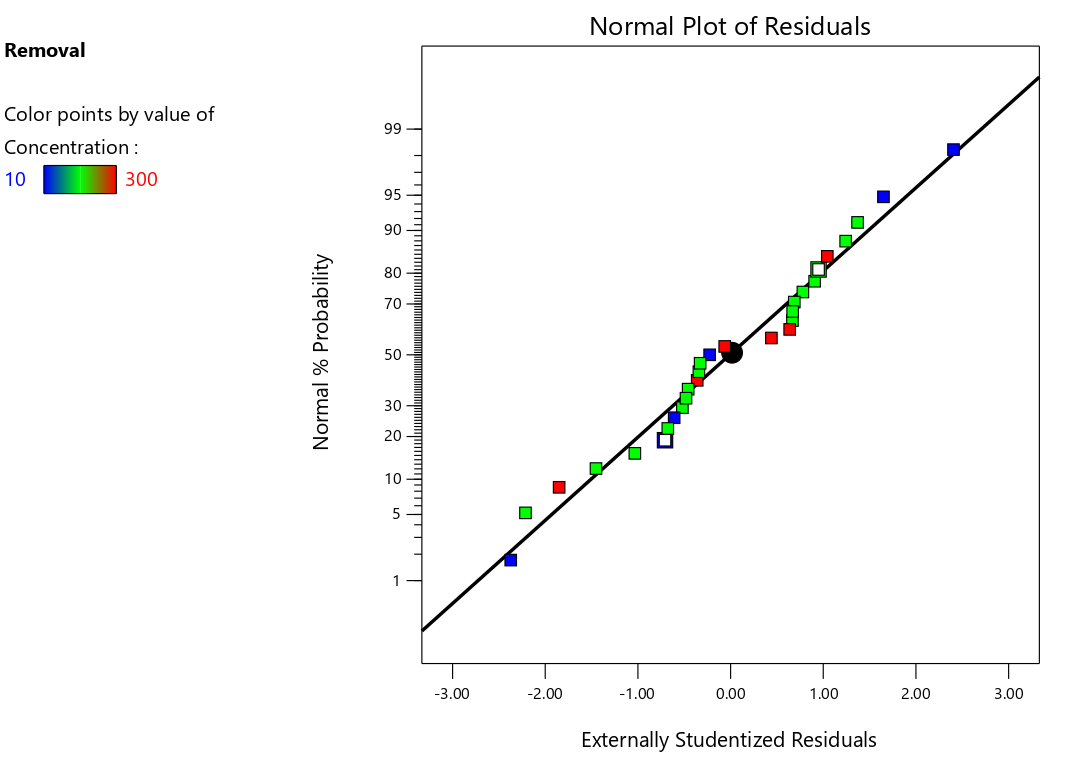


**Supplementary Figure 3.** Perturbation graphs, A)-the factors at center values and B)- the factors at optimum values.


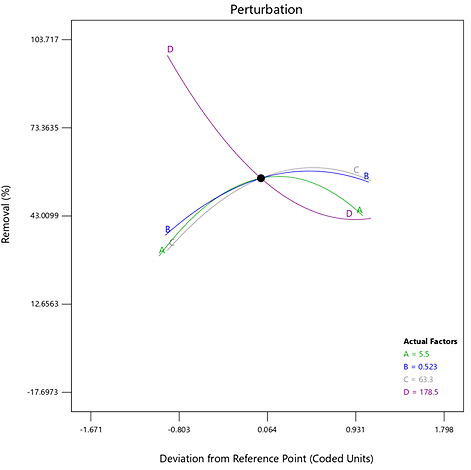


**A**


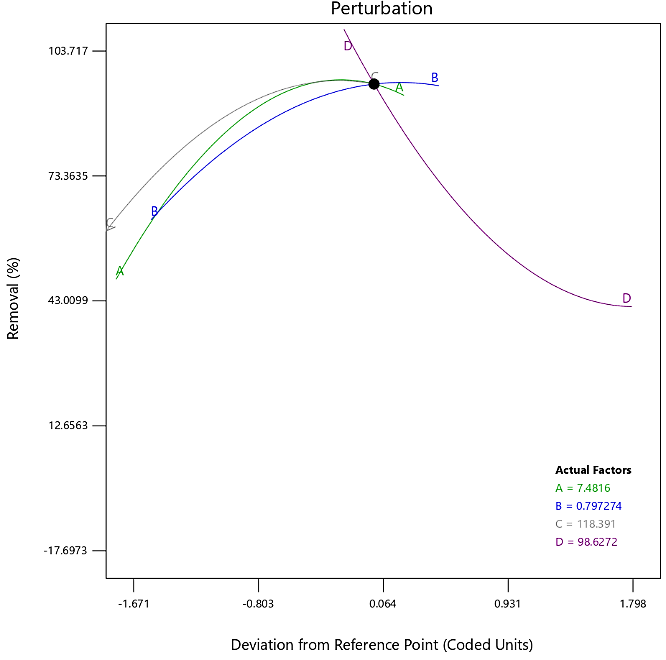


**B**
